# Mesenchymal stem cell therapy ameliorates metabolic dysfunction and restores fertility in a PCOS mouse model through interleukin-10

**DOI:** 10.1186/s13287-021-02472-w

**Published:** 2021-07-07

**Authors:** Rishi Man Chugh, Hang-soo Park, Abdeljabar El Andaloussi, Amro Elsharoud, Sahar Esfandyari, Mara Ulin, Lale Bakir, Alshimaa Aboalsoud, Mohamed Ali, Dalia Ashour, Prosper Igboeli, Nahed Ismail, Jan McAllister, Ayman Al-Hendy

**Affiliations:** 1 Department of Surgery, University of Illinois at Chicago, 820 South Wood Street, Chicago, IL 60612 USA; 2 Department of Radiation Oncology, University of Kansas Medical Center, Kansas City, KS 66160 USA; 3 Department of Obstetrics and Gynecology, University of Chicago, 5841 S. Maryland Ave, Chicago, IL 60637 USA; 4 Department of Pathology, University of Illinois at Chicago, 820 South Wood Street, Chicago, IL 60612 USA; 5 Department of Obstetrics and Gynecology, University of Illinois at Chicago, 820 South Wood Street, Chicago, IL 60612 USA; 6 Department of pharmacology, Faculty of Medicine, Tanta University, Tanta, Egypt; 7 Clinical Pharmacy Department, Faculty of Pharmacy, Ain Shams University, Cairo, Egypt; 8 Department of Pathology, Penn State Hershey College of Medicine, Hershey, PA USA

**Keywords:** Mesenchymal stem cells (MSCs), Cell transplantation, Cytokines, Interleukin

## Abstract

**Background:**

Polycystic ovary syndrome (PCOS) is the most common endocrine and metabolic disorder in reproductive-age women. Excessive inflammation and elevated androgen production from ovarian theca cells are key features of PCOS. Human bone marrow mesenchymal stem cells (BM-hMSC) and their secreted factors (secretome) exhibit robust anti-inflammatory capabilities in various biological systems. We evaluated the therapeutic efficacy of BM-hMSC and its secretome in both in vitro and in vivo PCOS models.

**Methods:**

For in vitro experiment, we treated conditioned media from BM-hMSC to androgen-producing H293R cells and analyzed androgen-producing gene expression. For in vivo experiment, BM-hMSC were implanted into letrozole (LTZ)-induced PCOS mouse model. BM-hMSC effect in androgen-producing cells or PCOS model mice was assessed by monitoring cell proliferation (immunohistochemistry), steroidogenic gene expression (quantitative real-time polymerase chain reaction [qRT-PCR] and Western blot, animal tissue assay (H&E staining), and fertility by pup delivery.

**Results:**

BM-hMSC significantly downregulate steroidogenic gene expression, curb inflammation, and restore fertility in treated PCOS animals. The anti-inflammatory cytokine interleukin-10 (IL-10) played a key role in mediating the effects of BM-hMSC in our PCOS models. We demonstrated that BM-hMSC treatment was improved in metabolic and reproductive markers in our PCOS model and able to restore fertility.

**Conclusion:**

Our study demonstrates for the first time the efficacy of intra-ovarian injection of BM-hMSC or its secretome to treat PCOS-related phenotypes, including both metabolic and reproductive dysfunction. This approach may represent a novel therapeutic option for women with PCOS. Our results suggest that BM-hMSC can reverse PCOS-induced inflammation through IL-10 secretion. BM-hMSC might be a novel and robust therapeutic approach for PCOS treatment.

**Supplementary Information:**

The online version contains supplementary material available at 10.1186/s13287-021-02472-w.

## Background

Polycystic ovary syndrome (PCOS) is the most common endocrine disorder affecting 15–18% of reproductive-age women [1, 2]. The disorder is characterized by hyperandrogenism, ovulatory dysfunction, and polycystic ovarian morphology [1]. Ovulatory dysfunction can lead to infertility and endometrial cancer [3, 4]. Many women with PCOS also exhibit metabolic aberrations such as insulin resistance, dyslipidemia, and hypertension, which are often worsened by concomitant abdominal adiposity or frank obesity. These factors can predispose women to abnormal glucose tolerance, cardiovascular disease, and even full-blown metabolic syndrome [5–7].

Women with PCOS exhibit chronic low-grade inflammation that can be perpetuated by ingestion of nutrients [8, 9]. In PCOS, molecular markers of inflammation triggered by glucose and saturated fat are highly correlated with insulin resistance and hyperandrogenemia [8–11]. Chronic androgen suppression does not decrease inflammation in PCOS [12], and pro-inflammatory stimuli are capable of increasing theca cell androgen production and the expression of enzymes responsible for producing androgens in vitro [13]. Recently, anti-inflammatory therapy has been shown to reduce ovarian androgen secretion and induce ovulation in lean, insulin-sensitive women with PCOS [14]. These findings clearly implicate inflammation as an underlying mechanism of ovarian dysfunction even in the absence of insulin resistance in PCOS.

In the last decade, extensive research has focused on the immunosuppressive and anti-inflammatory effects of bone marrow mesenchymal stem cells (BM-hMSC). Several reports suggest that these effects are mediated by secreted factors including interleukin (IL)-10, an anti-inflammatory cytokine [14–16]. These secreted factors are either released following cross-talk with target cells or produced constitutively by BM-hMSC [15, 17]. As chronic low-grade inflammation is strongly implicated as a driver of pathophysiology in PCOS [18], we hypothesized that interventions involving BM-hMSC or its secreted factors can improve the endocrine and metabolic abnormalities observed in PCOS, as well as fertility outcomes.

Mice receiving letrozole (LTZ), a nonsteroidal aromatase inhibitor, exhibit increases in circulating testosterone due to impaired conversion of testosterone to estrogen [19]. These mice exhibit the hallmarks of hyperandrogenism, anovulation, and polycystic ovaries, as well as impaired fertility and the metabolic dysregulation often observed in women with PCOS [19, 20]. Recently, anti-inflammatory therapy has been shown to reverse key features of the PCOS phenotype in this mouse model [11]. Thus, the LTZ-induced PCOS mouse is a useful model to evaluate the effect of BM-hMSC on inflammatory pathophysiological mechanisms in PCOS, and to establish novel stem cell-based therapeutics for PCOS. Because approximately half of women diagnosed with PCOS have excessive adrenal androgen production [21–23], androgen-producing human adrenocortical-carcinoma cells (H295R) represent a good in vitro model of PCOS.

We analyzed the effects of exposing H295R cells to the BM-hMSC secretome and IL-10 on testosterone production and the expression of genes encoding enzymes involved in androgen biosynthesis. We performed similar experiments on primary cultures of theca cells obtained from women with PCOS. Finally, we examined the effect of intra-ovarian injection of BM-hMSC on inflammation, metabolism, and ovarian and endometrial gene expression, as well as measures of fertility, in LTZ-induced PCOS mice.

## Methods

### Human bone-marrow mesenchymal stem cell culture

Human BM-hMSC (Passage 2) were purchased from Lonza, USA (PT#2501). These cells were isolated from the bone marrow of a healthy non-diabetic female donor 32-year-old. The cells were cultured in mesenchymal stem cell growth medium (MSCGM) per the manufacturer’s recommended expansion protocol. When the culture reached approximately 80% confluence, cells were trypsinized using a 0.05% trypsin-EDTA solution and serially expanded for use in experiments. Cells were characterized for typical BM-MSC-positive (CD90, CD73, CD105) and negative (CD34, CD11b, CD19, CD45, HLA-DR) cell surface markers using the human MSC analysis kit (BD Stemflow^TM^, CA, USA cat. no. 562245).

### Human adrenocortical-carcinoma cell line culture

Human adrenocortical-carcinoma cells (H295R cells) were used as an in vitro cell culture model for androgen production. These cells are commonly used in studies of steroidogenesis and androgen biosynthesis pathways [21–23]. H295R cells were purchased from ATCC (Manassas, VA, USA, cat. no. ATCC® CRL-2128™) and cultured per the recommended guidelines. Briefly, H295R cells were cultured in flasks pre-coated with extracellular matrix (Gibco, USA, cat. no. S-006-100) with DMEM/F12 (Gibco, cat. no. 21041025) and 2.5% Nu-Serum (Corning, USA). The cells were subcultured at a ratio of 1:3 to 1:4 and culture media were changed twice a week.

### Human theca cell culture from women with PCOS

We have obtained informed consents from the patients for human PCOS Theca cells. Human theca interna tissue was collected at the time of oophorectomy (*n*=2), which was performed as clinically indicated using a protocol [24–27] approved by the Institutional Review Board of The Pennsylvania State University College of Medicine. Theca cells from PCOS ovarian follicles were isolated and cultured as previously reported [28, 29]. The follicles were isolated from the ovaries and dissected under a microscope in a dish containing a 1:1 mixture of DMEM and Ham’s F12 medium supplemented with 10% fetal bovine serum (FBS). The cleaned theca shells were digested with 0.05% collagenase I, 0.05% collagenase IA, and 0.01% deoxyribonucleic phosphatase (DNase I), in medium containing 10% FBS. The isolated cells were cultured in dishes pre-coated with fibronectin in a 1:1 mixture of DMEM and Ham’s F12 medium containing 10% FBS, 10% horse serum, 2% UltroSer G, 20 nm insulin, 20 nm selenium, 1 μM vitamin E, and antibiotics. Experiments were performed using passage 4 (31–38 population doublings) PCOS theca cells.

### Preparation of the BM-hMSC secretome

The secretome was prepared from three to five passages of BM-hMSC in T75 flasks. Media were collected and discarded from the BM-hMSC culture at 80–90% confluence. Cells were then washed three times with phosphate-buffered saline (PBS) for complete removal of serum. Cells were then maintained in DMEM/F12 (Gibco, USA) serum-free media for 24 h to collect the secretome. After 24 h, the media were collected, centrifuged at 500*g* for 5 min at 4 °C to remove the cell debris, aliquoted, and stored at −80 °C for use in experiments. DMEM/F12 serum-free media without cells were incubated for 24 h in the T75 cell culture flask to serve as a negative control.

For in vivo experiments, the secretome was collected using the above method, and cultured cells were trypsinized from the flasks and counted. The average cell count was 2.25 × 10^6^ cells per flask. The collected BM-hMSC media were then aliquoted at a volume calculated based on the cell secretions from 5 × 10^5^ cells on average per ovary of each mouse. The media/secretome were concentrated using a vacuum concentrator (Labconco, MO, USA) and stored at − 80°C for use in in vivo experiments. Before intra-ovarian injection, the concentrated secretome was reconstituted with PBS to a final volume of 10 μl per ovary.

### Treatment of H295R cells and human PCOS theca cells with the BM-hMSC secretome

H295R cells and human PCOS theca cells were cultured separately on pre-coated six-well plates for 48 h. Cells were then treated for 24 h with secretome diluted in basal media (serum-free) at a 1:1 ratio. Cell culture media were replaced with serum-free media or secretome media, and cells were incubated for an additional 24 h. After the incubation period, cells were collected for analysis of steroidogenesis-related gene expression. Cell culture media was used for hormone quantification using an automated chemiluminescence immunoassay system, UniCel DxI 800, Access Immunoassay System (Beckman Coulter Inc., CA, USA) [30].

### Treatment of H295R cells with recombinant human IL-10

To investigate the anti-inflammatory effect of the BM-hMSC secretome, we measured the amount of IL-10 secreted by BM-hMSC into the culture media by ELISA (Abcam, Cambridge, MA, USA) following the manufacturer’s instructions [17]. We explored the effect of IL-10 on steroidogenesis-related gene expression, androgen secretion, and pro-inflammatory marker expression in H295R cells after treatment with 0, 125, 250, or 500 pg/ml recombinant human IL-10 (rhIL-10; R & D Biosystem, Cat No. 217-IL-010). These concentrations were selected based on the previously reported level of IL-10 secreted by hMSCs [31]. H295R cells were then collected for gene expression analysis, and cell culture media were used for measurement of testosterone using an automated chemiluminescence immunoassay system, UniCel DxI 800, Access Immunoassay System (Beckman Coulter Inc., CA, USA) [30] and androstenedione using ELISA (Biovision, CA, USA) [32].

### PCOS mouse model and intra-ovarian injection of BM-hMSC

Three-week-old female C57BL/6 mice (Charles River, MA, USA) were housed in a vivarium for 1 week under specific pathogen-free conditions. The animal experiment protocol for this study was approved by the University of Illinois at Chicago Animal Care Committee (UIC ACC). All animal experiments were performed in compliance with the University of Illinois at Chicago’s policies and guidelines for use of laboratory animals.

At 4 weeks of age, mice (n = 10/group) were subcutaneously implanted with a placebo or 5 mg LTZ pellet (Innovative Research of America, Sarasota, FL, USA), which provides a constant release of LTZ (50 μg/day). Body weight was monitored weekly before and post-implantation. Body weight and insulin resistance (measured by glucose tolerance test, GTT) were used to monitor development of PCOS characteristics.

Five weeks after placebo or LTZ pellet implantation, mice underwent intra-ovarian injection of BM-hMSC via laparotomy. Mice were treated preoperatively with a single dose of buprenorphine (0.1 mg/kg) and were kept under anesthesia with 1−4% inhalation of isoflurane during the entire procedure. A single midline incision, less than 25 mm, was made on the skin to access both ovaries via the caudal abdominal cavity. For the BM-hMSC group, cells were injected in both ovaries at a concentration of 5.0 × 10^5^ cells per ovary resuspended in 10 μl PBS. For the secretome group, concentrated secretome reconstituted in 10 μL PBS was injected per ovary in both ovaries. For the control group, 10 μl of PBS was injected into both ovaries. The incision was closed by suturing, followed by wiping with a clean disinfectant swab. Two weeks after BM-hMSC engraftment or secretome injection, the mice were anesthetized and gonadal fat pads, brown fat, and ovaries were collected. A portion of the gonadal and brown fat, as well as one ovary, were fixed in 4% paraformaldehyde and embedded in paraffin; the remainder of the tissue and the other ovary was frozen at -80°C for further analysis.

### Glucose tolerance test

Glucose tolerance testing was performed on mice 5 weeks after placebo or LTZ pellet implantation and 2 weeks after BM-hMSC engraftment or secretome treatment. Mice were fasted for 16 h (5 p.m. to 9 a.m.), with free access to drinking water, after which they received an intraperitoneal (i.p.) injection of D-glucose (2.0 g/kg body weight). Blood glucose level was measured at 0, 15, 30, 60, 90, and 120 min following glucose injection using a Bayer glucose monitor (Roche Diagnostics Corp, IN, USA).

### Indirect calorimetry

Metabolic rate was measured in mice at 11 weeks of age by indirect calorimetry in open-circuit Oxymax chambers, a unit of the Comprehensive Lab Animal Monitoring System (CLAMS; Columbus Instruments, state, USA). Two weeks after BM-hMSC treatment, mice receiving LTZ only or LTZ and treated with BM-hMSC (n=3) were acclimated to calorimetry cages for 2 days before data sampling at 23 °C under 12:12 h light to dark cycle. Oxygen consumption rate (VO_2_), carbon dioxide release (VCO_2_), respiratory exchange ratio (RER), and heat production were measured in individual mice. The horizontal activity was measured on x-, y-, and z-axes.

### Serum hormone measurements

Blood was collected from all the groups by cardiac exsanguination under isoflurane anesthesia; serum was separated and stored at − 80°C. Serum hormone levels were measured at the University of Virginia Ligand Core Facility. Serum testosterone (T) and estradiol (E2) levels were measured using ELISA. Serum luteinizing hormone (LH) and follicle-stimulating hormone (FSH) levels were measured by radioimmunoassay (RIA). The sensitivities of each assay are 10 ng/dL (T), 3 pg/ml (E2), 3 ng/ml (FSH), and 0.04 ng/ml (LH). Serum cytokines were analyzed in a membrane-based antibody array (Ray Biotech, GA, USA) per the manufacturer’s protocol.

### Breeding experiments

One week after BM-hMSC engraftment or secretome treatment, 6 mice per group were randomly selected for the breeding experiment. One male C57BL/6 breeder mouse was used for every two female mice. The male and female mice were caged together for 10 days. Mating was determined by the presence of sperm plug in the vagina. Most of the female mice showed a sperm plug within 3 days, and the average number of pups from each female mouse was compared between treatment groups. At the end of the experiment, all delivered pups were counted per group, their body weight was measured, and any morphological anomalies were noted.

### Histology and immunohistochemistry

Ovaries and fat tissues were collected, fixed in 4% paraformaldehyde, and embedded in paraffin blocks. Tissue sections were stained with hematoxylin-eosin (H&E) and murine anti-UCP-1 (Abcam, MA, USA), followed by detection with a biotin-labeled rabbit anti-rat antibody and staining with the ABC kit (Vector Laboratories, Burlingame, CA, USA). Sample processing and staining were performed at the histology core of the University of Illinois at Chicago (Chicago, IL, USA). Histological analyses were performed using Asperio ImageScope (Leica Biosystem, Wetzlar, Germany).

### Immunoblot analysis

Following treatment of H295R cells and human PCOS theca cells with the BM-hMSC secretome, and treatment of mice with BM-hMSC or its secretome, cultured cells and collected ovarian tissue were lysed with RIPA buffer (Cell Signaling, MA, USA) containing protease and phosphatase inhibitor cocktail (Thermo Fisher Scientific Inc., MA, USA) and sonicated at 20 amplitude with 5 s on and 5 s off for a 1-minute cycle. Sonicated samples were then centrifuged at 12,000 rpm for 5 min and the supernatant was transferred into separate tubes. The protein concentration of all samples was determined by the Bradford method. For immunoblot analysis, samples containing equal amounts of protein were incubated with 1x gel loading buffer and separated by SDS-PAGE (4–20% criterion, Bio-Rad), then transferred to PVDF membrane using a Trans-blot turbo system (Bio-Rad, Hercules, CA, USA). After protein transfer, blocked membranes were incubated in 1% non-fat dry milk in 1x PBS (0.05% Tween) overnight at 4 °C, with primary antibodies against CYP17A1 (ab125022, 1:500 dilution, Abcam), CYP11A1 (ab75497, 1:500 dilution, Abcam), DENND1A (LS-C167356, 1:250, LSBio), VEGFA (ab1316, 1:500 dilution, Abcam), or β-actin (clone AC-15, A5441, 1:5000, Sigma) in 1% non-fat dry milk in 1x PBS with 0.05% Tween overnight at 4°C. After washing, the membrane was incubated with the appropriate HRP-linked secondary antibodies (anti-mouse secondary antibody, cat. no. 7076, 1:5000 or anti-rabbit secondary antibody, cat. no. 7074, 1:3000, Cell Signaling) in 5% non-fat dry milk in 1x PBS with 0.1% Tween at room temperature for 1 h. The membrane was developed with Trident Femto Western HRP substrate (GeneTex, Irvine, CA, USA) and visualized using the ChemiDoc XRS + molecular imager (Bio-Rad, Hercules, CA, USA). After imaging, membranes were stripped with Restore^TM^ PLUS stripping buffer (Thermo Scientific, MA, USA) to incubate with another antibody. The signal density of each protein band was quantified using Image J software (US National Institute of Health, Bethesda, MD, USA) and normalized against the corresponding β-actin band.

### Quantitative RT-PCR

RNA was extracted from H295R cells and human PCOS theca cells treated with the BM-hMSC secretome or rhlL-10. RNA was also extracted from fat and ovarian tissues collected from mice treated with BM-hMSCs or the BM-hMSC secretome. RNA extraction was done using TRIzol (Invitrogen, USA) according to the manufacturer’s instructions. The concentration and purity of the extracted RNA were checked using a NanoDrop spectrometer (Thermo Scientific, MA, USA). One microgram of total RNA was reverse transcribed using RNA to cDNA EcoDry™ Premix (Double Primed) (Takara Bio USA Inc., CA, USA). The reaction mixture was incubated for 1 h at 42 °C; incubation was stopped at 70 °C for 10 min. Quantitative real-time PCR (qPCR) was performed using the CFX96 PCR instrument and SYBR Green Supermix (Bio-Rad, Hercules, CA, USA) with specific primers to the target genes in a 20-μL final reaction volume. The primer sequences are listed in Table S1. Beta-actin was used as a reference gene for sample normalization. The delta-delta threshold cycle (ΔΔCt) method was used to calculate the fold change expression in mRNA level in the samples.

### Flow cytometry (FACS) analysis

After treatment with the BM-hMSC secretome or basal media control, H295R cells were analyzed by FACS for proliferation, apoptosis, and inflammatory markers using antibodies against Ki67 antibody (BioLegend, Cat no. 350514), Annexin-V (BioLegend, Cat no. 640919), IL-1β (R&D Systems, Cat no. IC8406A), and TNF-α (BioLegend, Cat no. 502943). In brief, treated cell pellets were harvested and fixed/permeabilized with BD cytofix/cytoperm kit reagent (BD Bioscience, CA, USA) for intracellular staining, per the manufacturer’s instructions. After centrifugation at 1500 rpm for 5 min, a total of 1 × 10^6^ cells were resuspended in 200 μl of antibody solution and incubated for 30 min at room temperature in the dark. After washing, the cells were resuspended in PBS with 2% FBS (v/v) for FACS analysis using (BD, Gallios, Flow-cytometer). Data were analyzed using FlowJo software.

### Statistical analysis

Comparisons between groups were made by two-way ANOVA or nonparametric T-test (Mann-Whitney test) using GraphPad Prism 9 (GraphPad Software, San Diego, CA). All data are presented as mean ± standard deviation (SD). A difference between groups with ∗*p*<0.05, ∗∗*p*<0.005, or ∗∗∗*p*<0.0005 was considered statistically significant.

## Results

### BM-hMSC secretome elicits anti-proliferative and apoptotic effects in H295R cells

**Fig. 1:**
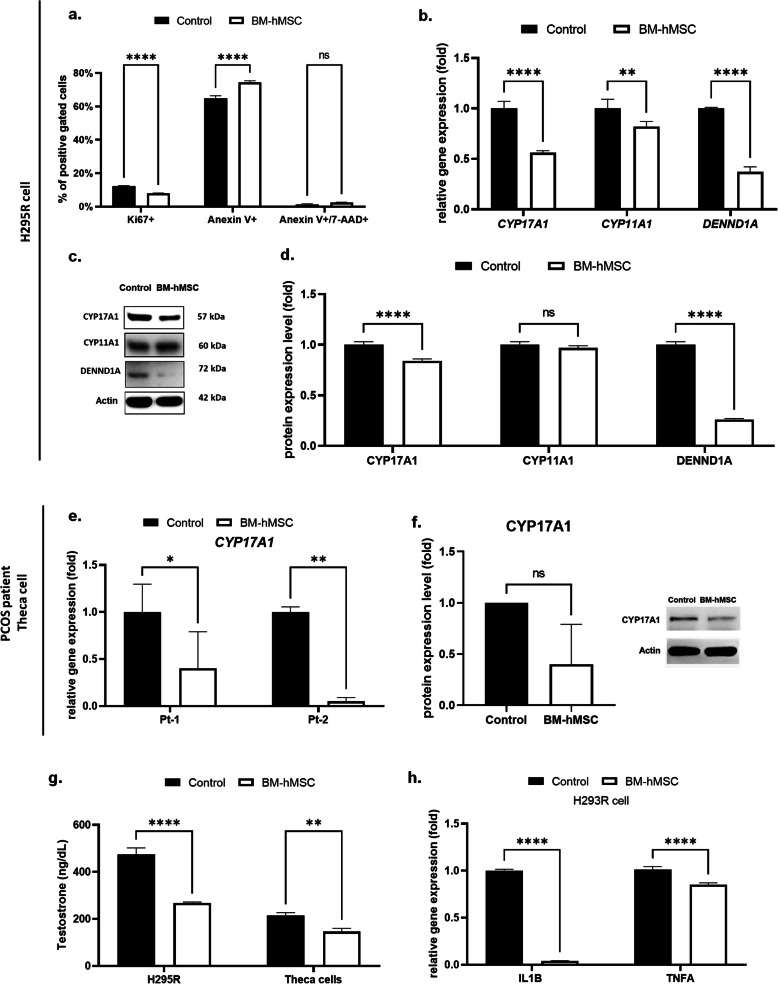
Effect of BM-hMSC secretome on H295R cells and human PCOS theca cells. After 24 hours of treatment, **a** percentage of Ki67-positive cells, Annexin V-positive cells, and Annexin V+/7AAD double-positive cells in the BM-hMSC secretome-treated vs. control (basal media) H295R cells. **b** Relative mRNA expression of *CYP17A1*, *CYP11A1*, and *DENND1A* in BM-hMSC secretome-treated vs. control H295R cells. **c** Protein expression analysis of CYP17A1, CYP11A1,and DENND1A by western blot in BM-hMSC secretome-treated vs. control H295R cells. **d** Quantification of western blot by analyzing band intensity. **e** Relative mRNA expression of *CYP17A1* in two different human PCOS theca cells after BM-hMSC secretome treatment vs. control (basal media). **f** Average protein expression level of CYP17A1 in human PCOS theca cells after BM-hMSC secretome treatment vs. control. **g** Testosterone secretion by H295R cells and human PCOS theca cells; BM-hMSC secretome vs. control group. **h** Relative gene expression of inflammatory marker *IL1B* and *TNFA* in BM-hMSC secretome-treated vs. control basal media in H295R cells. *p<0.05, **p<0.005, ***p<0.0005; NS, not significant. All graphs are presented as the mean ± SD (n=3). Statistical significances were determined by 2-way ANOVA or nonparametric T-test (Mann-Whitney test) using GraphPad Prism 9

H295R cells were incubated with BM-hMSC secretome to evaluate therapeutic potential. After 24 h, we observed a significant reduction (7.96% ± 0.23) in cell growth rate, as measured by Ki-67 protein expression compared with control media-treated cells (12.37% ± 0.19; Fig. 1a). Additionally, secretome treatment significantly increased both early apoptosis (74.38% ± 1.00; Fig. 1a) as well as late apoptosis and necrosis (2.43% ± 0.21; Fig. 1a), as measured by Annexin-V and Annexin-V/7-AAD expression, respectively, compared with the control group (64.94% ± 1.47 and 1.30% ± 0.43). Thus, our results indicate that BM-hMSC secretome inhibits growth of H295R cells.

### BM-hMSC secretome decreases steroidogenesis-related gene expression and androgen production in H295R cells

We previously reported that *CYP17A1*, *CYP11A1*, and *DENND1A*, key genes for ovarian androgen biosynthesis, are upregulated in PCOS-theca cells compared with healthy theca cells [27, 33]. Hence, we evaluated the effect of the BM-hMSC secretome on the expression of these genes using our in vitro model. Secretome treatment resulted in significant downregulation of *CYP17A1* (0.56 ± 0.02 fold) and *DENND1A* (0.37 ± 0.05 fold) gene expression in H295R cells compared with media-treated cells (Fig. 1b). However there was no significant decrease in *CYP11A1* gene expression (0.82 ± 0.05 fold, *p*=0.127). We confirmed these findings at the protein level using immunoblot analysis, which showed that CYP17A1 (0.84 ± 0.02 fold) and DENND1A (0.26 ± 0.01 fold) were significantly decreased in secretome-treated H295R cells compared with the control group, while no change was observed in CYP11A1 (0.97 ± 0.02 fold, *p*=0.41; Fig. 1c, d). We validated these observations in PCOS patient-derived theca cells (n=2) treated with BM-hMSC secretome. Secretome treatment significantly downregulated CYP17A1 (Patient 1: 0.36 ± 0.20 fold, Patient 2: 0.05 ± 0.04 fold) gene expression (Fig. 1e) and shows decreasing trend of protein expression (0.40 ± 0.39 fold, *p*=0.1; Fig. 1f) in theca cells from both patients compared with media-treated controls.

We investigated the effects of steroidogenesis-related gene inhibition by the BM-hMSC secretome on testosterone secretion. We explored whether steroidogenesis-related gene inhibition by the BM-hMSC secretome affected testosterone secretion. Compared with the media control group (474.6±27.5 ng/dL), secretome treatment suppressed testosterone secretion in H295R cells (267.7±4.0 ng/dL) (Fig. 1g). Testosterone secretion was also suppressed in human PCOS theca cells (146.4±13.4 ng/dL) (Fig. 1g) compared with the media control group (214.7±11.8 ng/dL). In summary, our data indicate that BM-hMSC secreted factors inhibit androgen production.

### BM-hMSC secretome exerts an anti-inflammatory effect on H295R cells

Chronic inflammation is a major factor affecting the ovarian microenvironment in patients with PCOS inducing higher ovarian androgen production [34–37], which involves two pro-inflammatory cytokines, interleukin-1 beta (IL-1β), and tumor necrosis factor (TNF-α) [38]. Treatment of H295R cells with BM-hMSC secretome significantly downregulated gene expression of IL-1β (*IL1B*: 0.04 ± 0.003 fold) and TNF-α (*TNFA*: 0.85 ± 0.02 fold) compared with the control media group (Fig. 1h), indicating a decreased inflammatory response after treatment.

### IL-10 decreases steroidogenesis-related gene expression and androgen production in H295R cells

**Fig. 2:**
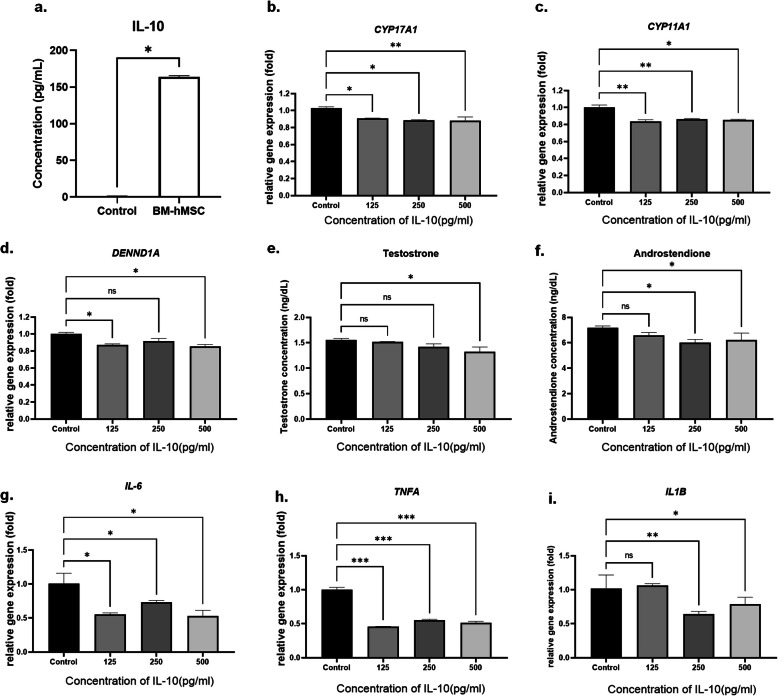
Effect of IL-10 on H295R cells. **a** Concentration of IL-10 secreted by BM-hMSC presented as the mean ± SD (n=4). Relative gene expression of **b** *CYP17A1*, **c** *CYP11A1*, and **d** *DENND1A* after IL-10 treatment. **e** Testosterone secretion and **f** androstenedione secretion by H295R cells after IL-10 treatment. Relative gene expression of inflammatory markers **g** IL-6 (*IL6*), **h** TNF-α (*TNFA*), and **i** IL-1β (*IL1B*) after IL-10 treatment. *p<0.05, **p<0.005; NS, not significant. **b**–**i** All graphs are presented as the mean ± SD (n=3). Statistical significances were determined by 2-way ANOVA or nonparametric T-test (Mann-Whitney test) using GraphPad Prism 9

Based on the observed anti-inflammatory effect of the BM-hMSC secretome, we tested the effect of the anti-inflammatory cytokine, IL-10, which is known to be released by MSC [31, 39, 40]. IL-10 exerts immune-suppressive and anti-inflammatory effects in several disorders, including PCOS [31, 41]. Recent reports highlighted a significantly lower serum level of IL-10 in women with PCOS compared with age- and BMI-matched healthy controls [42]. High IL-10 levels may also increase insulin sensitivity by ameliorating the inflammatory responses to TNF-α and IL-6, which contribute to insulin resistance in PCOS [43, 44]. First, we explored IL-10 secretion from BM-MSCs by ELISA of conditioned media. We found a high concentration of IL-10 (164.2±1.42 pg/ml) compared with control media (1.32±0.15 pg/ml; Fig. 2a). We then examined the effect of IL-10 on steroidogenesis-related gene expression and androgen production in H295R cells. As shown in Fig. 2, recombinant human IL-10 treatment significantly downregulated the expression of *CYP17A1* gene expression even in 125 pg/ml of concentration (0.91 ± 0.01 fold) (Fig. 2b). The same IL-10 treatment (125 pg/ml) condition also significantly decreased *CYP11A1* gene expression (0.84 ± 0.02 fold) and DENND1A gene expression (0.87 ± 0.01 fold) in H295R cells (Fig. 2c, d). Additionally, we also analyzed testosterone level in conditioned media from IL-10 treated H295R cells. Unfortunately, testosterone level was not significantly decreased in 125 pg/ml of IL-10-treated H295R cells (1.52 ± 0.01 ng/ml, *p*=0.789) compared to control (1.56 ± 0.02 ng/ml). However, in higher concentration of IL-10 (125 pg/ml) treatment was significantly decreased testosterone level in H295R cells (1.32 ± 0.10 ng/ml, *p*=0.02) (Fig. 2e). The androstenedione (Control: 1.55 ± 0.03 ng/ml) also did not decreased significantly in 125 pg/ml of IL-10 treated H295R cells (1.44 ± 0.04 ng/ml, p=0.15), but significantly decreased in higher concentration (250 pg/ml: 1.34 ± 0.04 ng/ml, 500 pg/ml: 1.38 ± 0.10 ng/ml) (Fig. 2f). Our data suggest that IL-10 inhibits androgen production by regulating steroidogenic gene expression.

### IL-10 exerts an anti-inflammatory effect on H295R cells

Next, we explored the anti-inflammatory effects of IL-10 on H295R cells by measuring expression of key pro-inflammatory cytokines, IL-6, TNF-α, and IL-1β, following IL-10 treatment. All tested concentrations of IL-10 significantly downregulated *IL6* (Control: 1.00 ± 0.08 fold, 125 pg/ml: 0.73 ± 0.03 fold, 250 pg/ml: 0.79 ± 0.02 fold, 500 pg/ml: 0.70 ± 0.11 fold), *TNFA* (Control: 1.00 ± 0.04 fold, 125 pg/ml: 0.44 ± 0.02 fold, 250 pg/ml: 0.59 ± 0.07 fold, 500 pg/ml: 0.54 ± 0.04 fold), and *IL1B* (Control: 1.00 ± 0.04 fold, 125 pg/ml: 0.84 ± 0.02 fold, 250 pg/ml: 0.60 ± 0.08 fold, 500 pg/ml: 0.76 ± 0.1 fold) gene expression compared with untreated controls (Fig. 2g–i). Together, these data suggest that IL-10 is a key mediator of the effect of the BM-hMSC secretome on in vitro human cell PCOS models.

### BM-hMSC reverse the metabolic phenotypes in an LTZ-induced PCOS mouse model

**Fig. 3:**
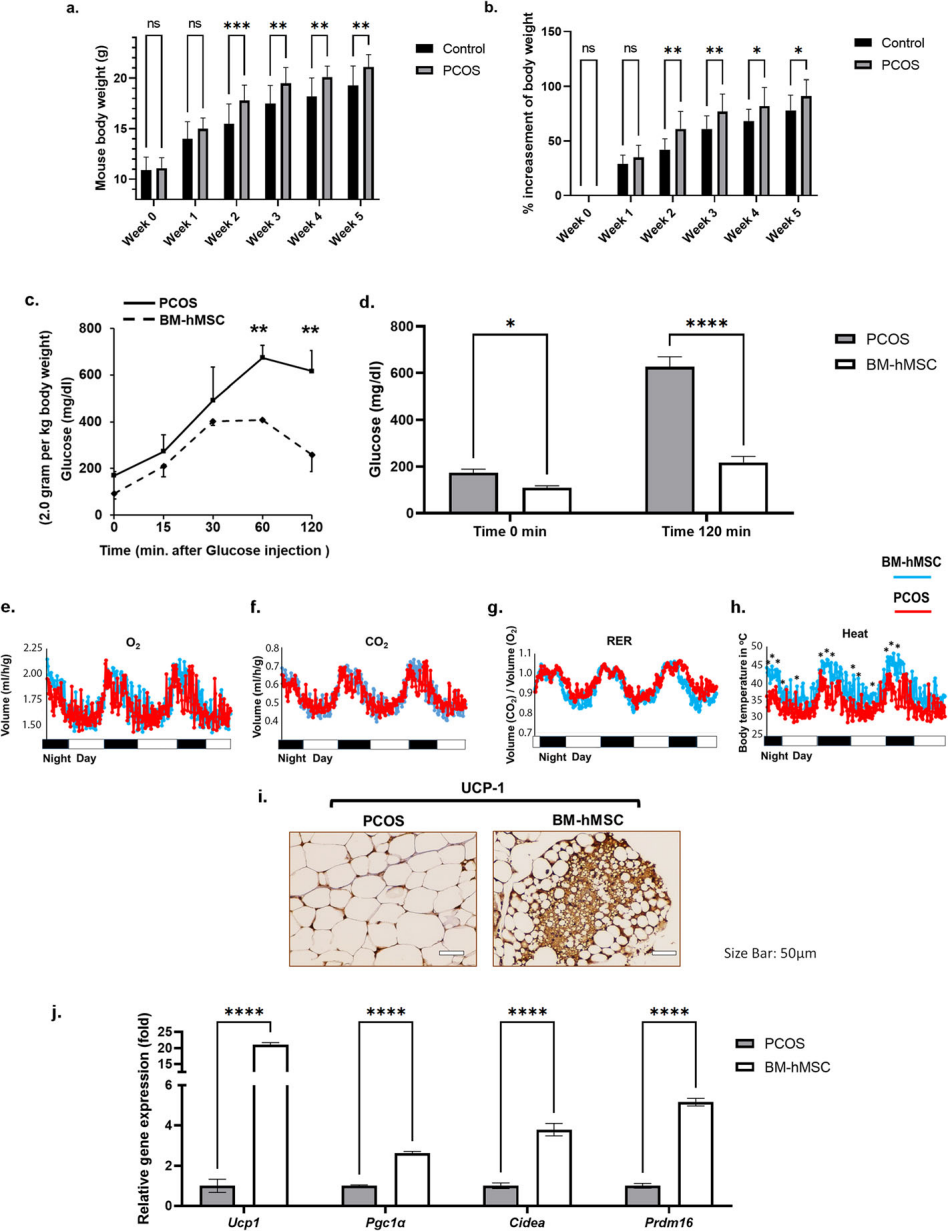
BM-hMSC injection into the ovary reverses metabolic phenotypes in the LTZ-induced PCOS mouse model. **a** Effect of LTZ on body weight in LTZ-treated mice (PCOS) and matched controls. Mean body weight after week 5 was significantly higher in the PCOS group (n=24) compared with matched controls (n=9). **b** Percent rate of increase in body weight in control mice (n=9) and PCOS mice (n=24). Graphs are presented as the mean ± SD. **c** Glucose tolerance test was performed on starved mice and mice after intraperitoneal (i.p.) glucose injection, monitored at the indicated time points for blood glucose level represented in mg/dL. **d** Blood glucose level at time 0 min and 120 min after glucose i.p. injection. BM-hMSC enhances energy expenditure in the PCOS mouse model. **e** Oxygen (O_2_), **f** carbon dioxide (CO_2_), **g** respiratory exchange ratio (RER), and **h** heat production presented in histograms comparing energy expenditure profiles of the PCOS group and BM-hMSC-treated PCOS group. **i**–**j** BM-hMSC induces browning of white fat in the PCOS mouse model. **i** UCP-1 immunohistochemistry staining of white gonadal fat. Scale bar is 50μm. **j** Relative gene expression of UCP-1 (*Ucp1*), PGC-1α (*Pgc1a*), *Cidea*, and *Prdm16* in white fat from PCOS mice and BM-hMSC-treated PCOS mice. *p<0.05, **p<0.005. **c**–**i** All graphs are presented as the mean ± SD (n=3). Statistical significances were determined by 2-way ANOVA using GraphPad Prism 9

Next, we evaluated the potential therapeutic effects of BM-hMSC in vivo by injecting BM-hMSC into the ovaries of the well-established LTZ-induced PCOS mouse model [19]. Five weeks after LTZ implantation, the mice in the PCOS group were significantly heavier (21.1 ± 0.25 grams) compared with age-matched control mice (19.3 ± 0.60 grams) that had received placebo pellets (Fig. 3a, b). Since PCOS women have insulin resistance and impaired glucose tolerance [45], we also performed a glucose tolerance test (GTT) and measured energy expenditure in PCOS mice before (5 weeks after LTZ or placebo) and 2 weeks after BM-hMSC engraftment (7 weeks after LTZ or placebo). Interestingly, PCOS mice treated with BM-hMSC exhibited a normal glucose tolerance profile compared with untreated PCOS mice (Fig. 3c, d). Moreover, we found that the untreated PCOS group had lower energy expenditure, based on a significant difference in thermogenesis, compared with PCOS mice treated with BM-hMSC (Fig. 3e–h).

The increase in thermogenesis in BM-hMSC-treated PCOS mice encouraged us to further evaluate fat metabolism in treated versus control PCOS mice. A previous study revealed the role of brown fat cells in the regulation of total energy expenditure [46]. A process called “browning,” which refers to the transition of white fat into brown fat, is associated with upregulation of UCP-1 [47]. Therefore, we stained white fat tissues collected from BM-hMSC-treated and untreated PCOS mice with UCP-1 and found greater proportions of brown-like fat cells, suggesting increased browning of white fat, in the BM-hMSC-treated group (Fig. 3i). At the molecular level, the browning process is regulated by several genes that control multiple aspects of mitochondrial activity, such as *Pgc-1α*, *Cidea*, and *Prdm-16* [48]. qPCR confirmed our UCP-1 immunohistochemistry results and showed significant upregulation of *Ucp1* (21.00 ± 0.67 fold), *Pgc1a* (2.61 ± 0.08 fold), *Cidea* (3.78 ± 0.31 fold), and *Prdm16* (5.15 ± 0.19 fold) gene expression in white fat collected from the BM-hMSC-treated PCOS mice compared with the untreated PCOS mice (Fig. 3j).

We also explored marker expression levels in brown fat tissue and found that BM-hMSC treatment increases brown fat-related marker expression even in brown fat tissue (Fig S4). These results suggest that BM-hMSC can regulate adipose tissue metabolism by ameliorating inflammation and promoting brown fat formation.

### BM-hMSC normalize the adipokine profile in adipose tissue in an LTZ-induced PCOS mouse model

Weight gain associated with LTZ-induced PCOS is partially due to white fat expansion [19]. The expansion of gonadal fat in our LTZ-induced PCOS mice was marked by characteristic morphologic enlargement of fat cells detected by H&E staining (Fig S5a). Remarkably, the average size of adipocytes in the BM-hMSC-treated PCOS mice was significantly smaller than that in the untreated PCOS mice (Fig S5b), approaching the normal size range of adipocytes.

White fat adipocyte expansion is usually associated with an increase in leptin that correlates inversely with adiponectin levels [49]. Studies have shown that adiponectin is a pivotal adipokine that can reverse PCOS metabolism [50], acting as a humoral factor that regulates fat homeostasis by establishing cross-talk between white and brown fat cells [51]. To explore such cross-talk in our PCOS mouse model, we measured gene expression of leptin and adiponectin in brown adipose tissue as well as white gonadal fat using qPCR. Treatment with BM-hMSC upregulated adiponectin and downregulated leptin expression, thus normalizing the ratio of leptin to adiponectin in brown fat tissue compared with the untreated group (Fig S5c-e). Similar findings were observed in the white gonadal fat (Fig S5f-h), highlighting the ability of BM-hMSC to normalize fat metabolism in our PCOS mouse model.

### Serum hormone analysis

To assess the endocrine status following BM-hMSC engraftment, total serum hormone levels in BM-hMSC-treated and untreated PCOS animals were measured. Serum T levels were significantly higher in the untreated PCOS group versus healthy controls, with no significant difference in the BM-hMSC-treated group (*p*=0.797). Furthermore, there were no changes in serum estrogen levels among the three groups. However, LH was significantly lower in the PCOS group than healthy controls, and LH levels decreased after BM-hMSC engraftment in PCOS mice. In addition, FSH levels were lower in the PCOS group compared with healthy controls and increased after BM-hMSC treatment, though the change was not statistically significant (Fig S6a-d).

### BM-hMSC treatment reverses endometrial abnormalities in an LTZ-induced PCOS mouse model

PCOS imparts abnormalities in endometrial tissue, such as the thickening of endometrium epithelial cells and aberration of steroid receptor gene expression [52–54]. Consequently, we analyzed endometrial tissue in BM-hMSC-treated versus untreated PCOS mice. The endometrial tissue of the PCOS group showed abnormal thickness (Fig S7a) and the AIB1 gene, known to be elevated in the PCOS endometrium [54], showed significant alterations in the PCOS group endometrium. These abnormalities were reversed in the BM-hMSC-treated PCOS group (Fig S7b). Similarly, steroid receptor genes AR and ERβ trended higher in the PCOS group and this was reversed after BM-hMSC treatment (Fig S7c, d).

Interestingly, the proliferation marker Ki-67 was significantly upregulated in BM-hMSC-treated PCOS mice compared with the untreated PCOS group (Fig S7e). Additionally, several inflammatory regulator genes such as IL6, IL16, CCL2, and TNF-α were higher in the PCOS group endometrium compared with normal control endometrium, and these changes were significantly reversed after BM-hMSC treatment (Fig S7f-i). These results suggest that intra-ovarian injection of BM-hMSC reversed various alterations in PCOS endometrium, at least partially, by normalizing steroid hormone receptors and inflammatory cytokine gene expression. These changes likely improved the quality of endometrium and contributed to the improved reproductive outcomes in the PCOS mice after BM-hMSC treatment.

### BM-hMSC restore fertility in an LTZ-induced PCOS mouse model

**Fig. 4:**
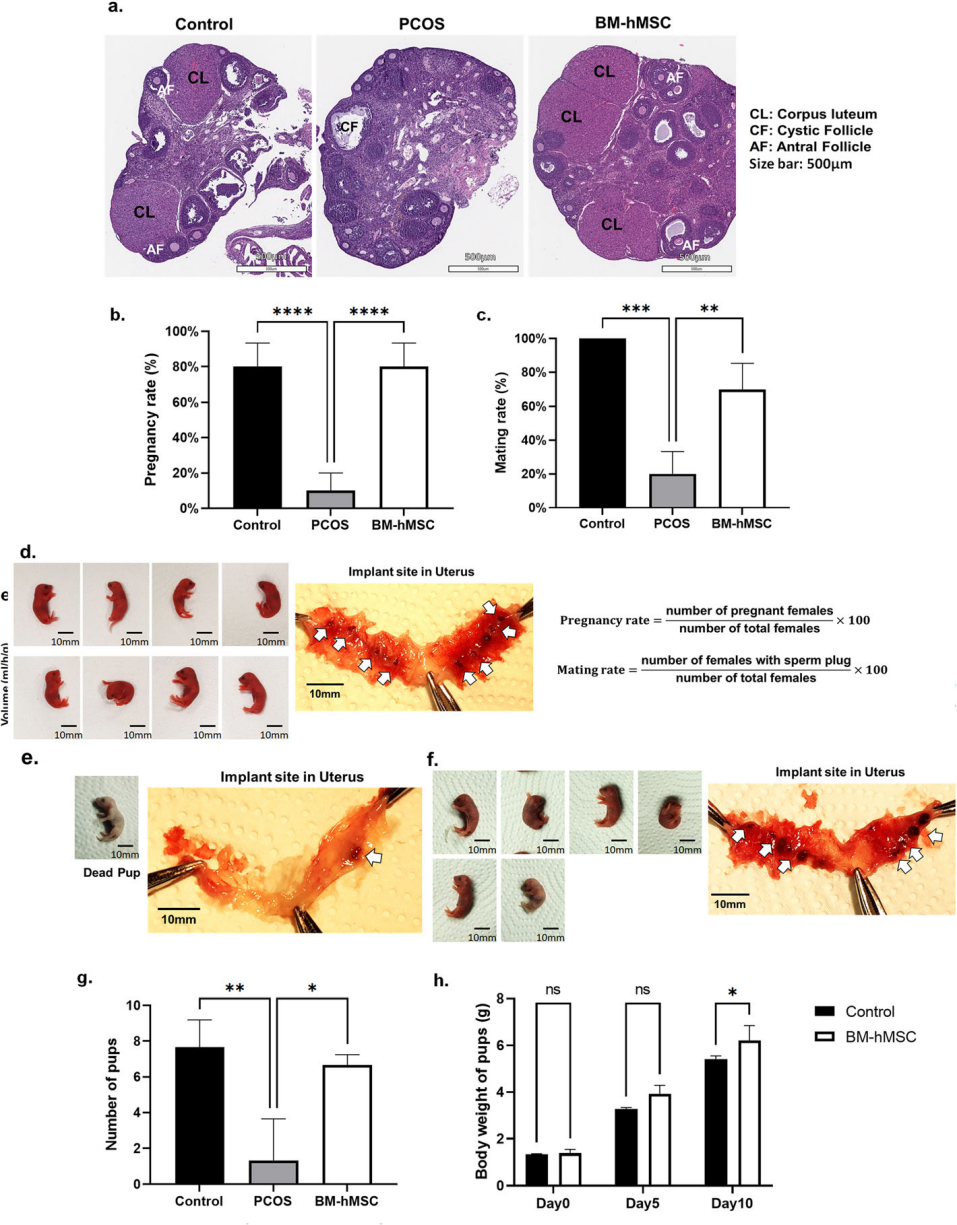
BM-hMSC injection into the ovary restores fertility in LTZ-induced PCOS mouse model. **a** Morphology of ovary from a normal mouse (control), LTZ-induced PCOS mouse (PCOS), and BM-hMSC-treated PCOS mouse (BM-hMSC). Scale bar is 500μm. **b** Pregnancy rate of the control group (8 out of 10), PCOS group (1 out of 10), and BM-hMSC-treated group (8 out of 10). Graphs are presented as the mean ± SEM (n=10). **c** The mating index (Mating rate) of the control group, PCOS group, and BM-hMSC-treated group. Graphs are presented as the mean ± SEM (n=10). Morphology of pups and implantation site in the uterus of **d** control group, **e** PCOS group, and **f** BM-hMSC-treated group. **g** Average number of pups from the control group, PCOS group, and BM-hMSC group. Graphs are presented as the mean ± SD (n=3). **h** Average body weight of pups post-natal day 10. Graphs are presented as the mean ± SD (n=3). *p<0.05, ***p<0.0005; NS, not significant. Statistical significances were determined by 2-way ANOVA using GraphPad Prism 9

To explore the effect of BM-hMSC treatment on reproductive function, we first analyzed ovarian morphology in BM-hMSC-treated versus untreated PCOS mice. Ovaries from untreated PCOS mice displayed typical PCOS characteristics, including lack of corpora lutea and antral follicles compared with untreated normal control mice (Fig. 4a). After intra-ovarian engraftment of BM-hMSC in both ovaries of PCOS mice, normal ovarian morphology was partially restored, including the reappearance of corpora lutea and antral follicles, as well as well-ordered stroma that was morphologically similar to that of the normal control group. These morphological changes suggest that BM-hMSC engraftment improved the pathological changes in PCOS ovaries and may potentially restore ovulation in PCOS mice (Fig. 4a).

Next, we performed a breeding experiment to test BM-hMSC’s treatment capacity to restore fertility in our PCOS mouse model. We found that healthy control mice had a higher rate of fertility (80%) than the subfertile PCOS group (10%). Interestingly, the pregnancy rate in BM-hMSC-treated PCOS mice was restored to a rate equal to that of the control group (Fig. 4b).

We also counted the number of delivered pups in all experimental groups. As shown in Fig. 4d–g, we found that most PCOS mice were infertile. The number of pups delivered in the BM-hMSC-treated PCOS group was equivalent to the number delivered in the control group. Moreover, the average number of pups delivered per mouse in the BM-hMSC-treated PCOS group (5.5 ± 1.1) was significantly higher than that in the untreated PCOS group (0.8 ± 1.7; Fig. 4g). We also found no significant differences in the average body weight of the delivered pups between the control group and BM-hMSC-treated PCOS group at 0, 5, and 10 postnatal days (Fig. 4h). Notably, we did not observe any apparent morphological abnormalities in any pups during the study period. We further tested the effect of injecting the BM-hMSC secretome in our PCOS mouse model (Fig S8). Fertility of PCOS mice was restored in secretome-treated animals. These results suggest that either BM-hMSC or its secretome can restore impaired fertility in an LTZ-induced PCOS mouse model with no detectable abnormalities in the delivered newborns.

### BM-hMSC restore normal ovarian gene expression in an LTZ-induced PCOS mouse model

**Fig. 5:**
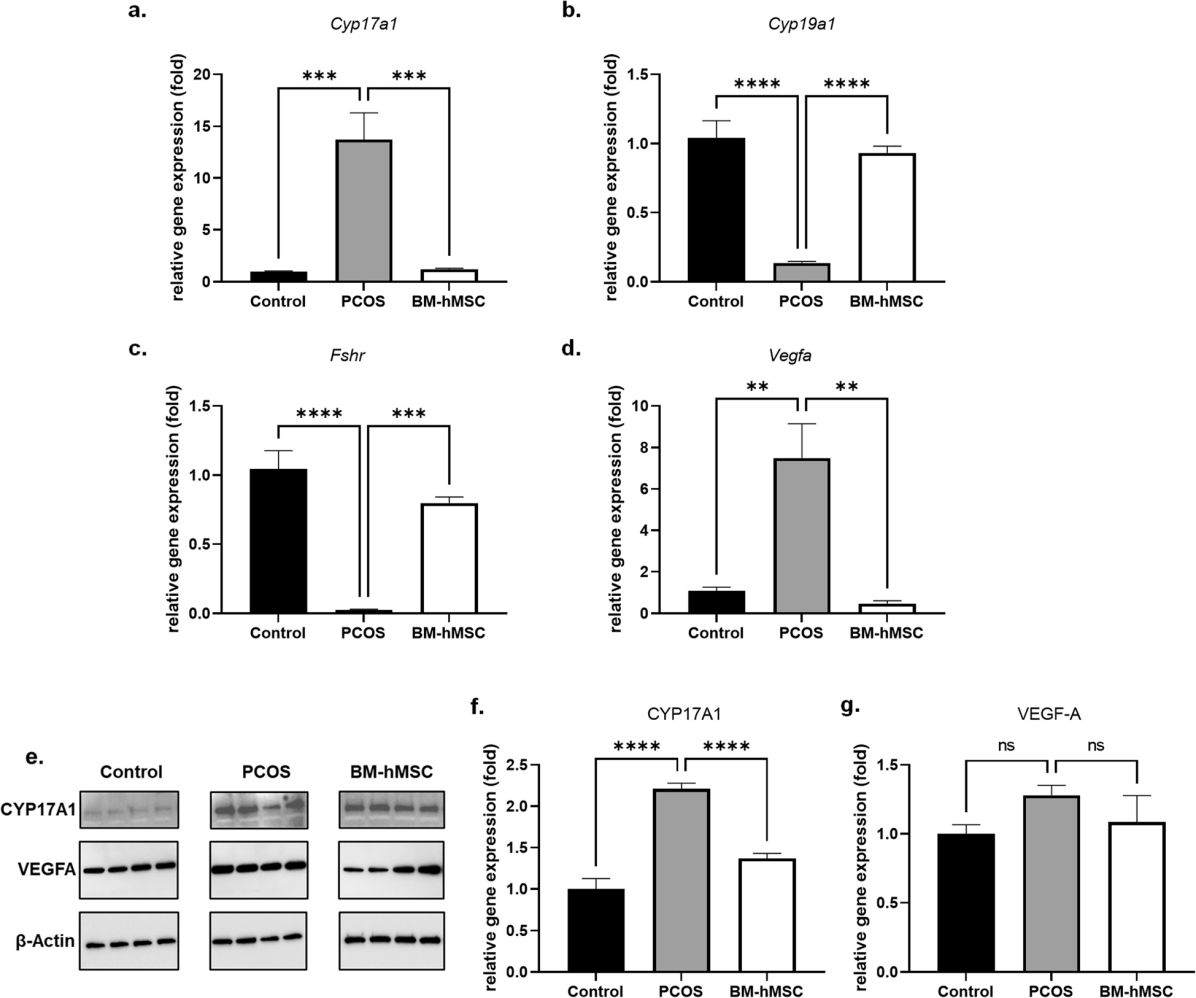
BM-hMSC injection into the ovary reverses altered gene expression in ovarian tissue of LTZ-induced PCOS mice. Relative gene expression of **a** *Cyp17a1*, **b** *Cyp19a1*, **c** *Fshr*, and **d** *Vegfa* in the ovary from control, untreated PCOS, and BM-hMSC-treated PCOS mice. **e** Immunoblot of CYP17A1 and VEGFA in the ovary. Quantification of **f** CYP17A1 and **g** VEGFA in the ovary from control, PCOS, and BM-hMSC-treated PCOS mice. *p<0.05, **p<0.005, ***p<0.0005; NS, not significant. All graphs are presented as the mean ± SD (n≥3). Statistical significances were determined by 2-way ANOVA or nonparametric T-test (Mann-Whitney test) using GraphPad Prism 9

PCOS abnormalities include enhanced androgen production and altered ovarian angiogenesis [55]. We next examined the effect of BM-hMSC treatment on these abnormalities in our PCOS mice in vivo, to validate our in vitro data on the secretome effects on ovarian steroidogenic gene and inflammation marker expression. Mouse ovarian tissues from BM-hMSC-treated and untreated animals were analyzed for RNA and protein levels of steroidogenesis and angiogenesis markers. *Cyp17a1* gene expression was significantly elevated in PCOS ovaries (13.73 ± 5.78 fold), which was significantly reversed after BM-hMSC treatment (1.22 ± 0.20 fold; Fig. 5a). *Cyp19a1* (0.14 ± 0.02 fold) and *Fshr* (0.03 ± 0.01 fold) gene expression were lower in PCOS group ovaries, consistent with the prior characterization of the LTZ-induced PCOS mouse model [19]; levels of both genes significantly increased after BM-hMSC treatment (*Cyp19a1*: 0.93 ± 0.11 fold, *Fshr*: 0.80 ± 0.09 fold; Fig. 5b, c). Previous studies reported an abnormal increase in ovarian angiogenesis in PCOS [55, 56]; thus, we measured gene expression of angiogenesis marker *Vegfa* in untreated and BM-hMSC-treated PCOS mice. Gene expression of *Vegfa* was elevated in the PCOS group (7.50 ± 3.69 fold) and decreased after BM-hMSC treatment (0.48 ± 0.29 fold; Fig. 5d). Immunoblot analysis supported these results, where CYP17A1 was higher in the PCOS group (2.21 ± 0.14 fold) and significantly decreased after BM-hMSC treatment (1.37 ± 0.12 fold; Fig. 5e, f). Moreover, VEGF-A protein expression was elevated in PCOS mice (1.28 ± 0.15 fold) and decreased after BM-hMSC treatment (1.08 ± 0.38 fold), although the change was not statistically significant (Fig. 5e, g). Taken together, our in vivo results suggest that BM-hMSC treatment can inhibit androgen synthesis and angiogenesis consistent with the effect of the BM-hMSC secretome on H295R cells.

### BM-hMSC secretome improves metabolic and reproductive phenotypes in LTZ-induced PCOS mice

Our in vitro and in vivo data suggest that the favorable effects of BM-hMSC engraftment likely occur in a paracrine fashion via secreted humoral factors in the BM-hMSC secretome. To explore the paracrine effect of BM-hMSC in the LTZ-induced PCOS mouse model, we delivered the BM-hMSC secretome by direct intra-ovarian injection into the ovaries of mice, and various metabolic and reproductive parameters were assessed. Analysis of white fat demonstrated a significant reduction in the size of fat cells in the secretome-treated PCOS group compared with the untreated PCOS group (Fig S8a), and the expression of UCP1 in brown fat tissue was also significantly higher in the secretome-treated PCOS group compared with untreated PCOS group (Fig S8b).

Morphological comparison of ovaries among the groups of mice by H&E staining revealed that secretome-treated PCOS ovaries had more antral follicles compared with untreated PCOS group ovaries (Fig S8c). Importantly, secretome treatment also restored fertility in PCOS mice compared with the untreated PCOS control group (Fig S8d, e). These results confirm the ability of the BM-hMSC secretome to reverse metabolic and reproductive abnormalities in the LTZ-induced PCOS mouse model and suggest that the positive effects of BM-hMSC are primarily mediated via paracrine action of the BM-hMSC secretome.

### BM-hMSC regulate inflammation via IL-10 in the LTZ-induced PCOS mouse model

**Fig. 6:**
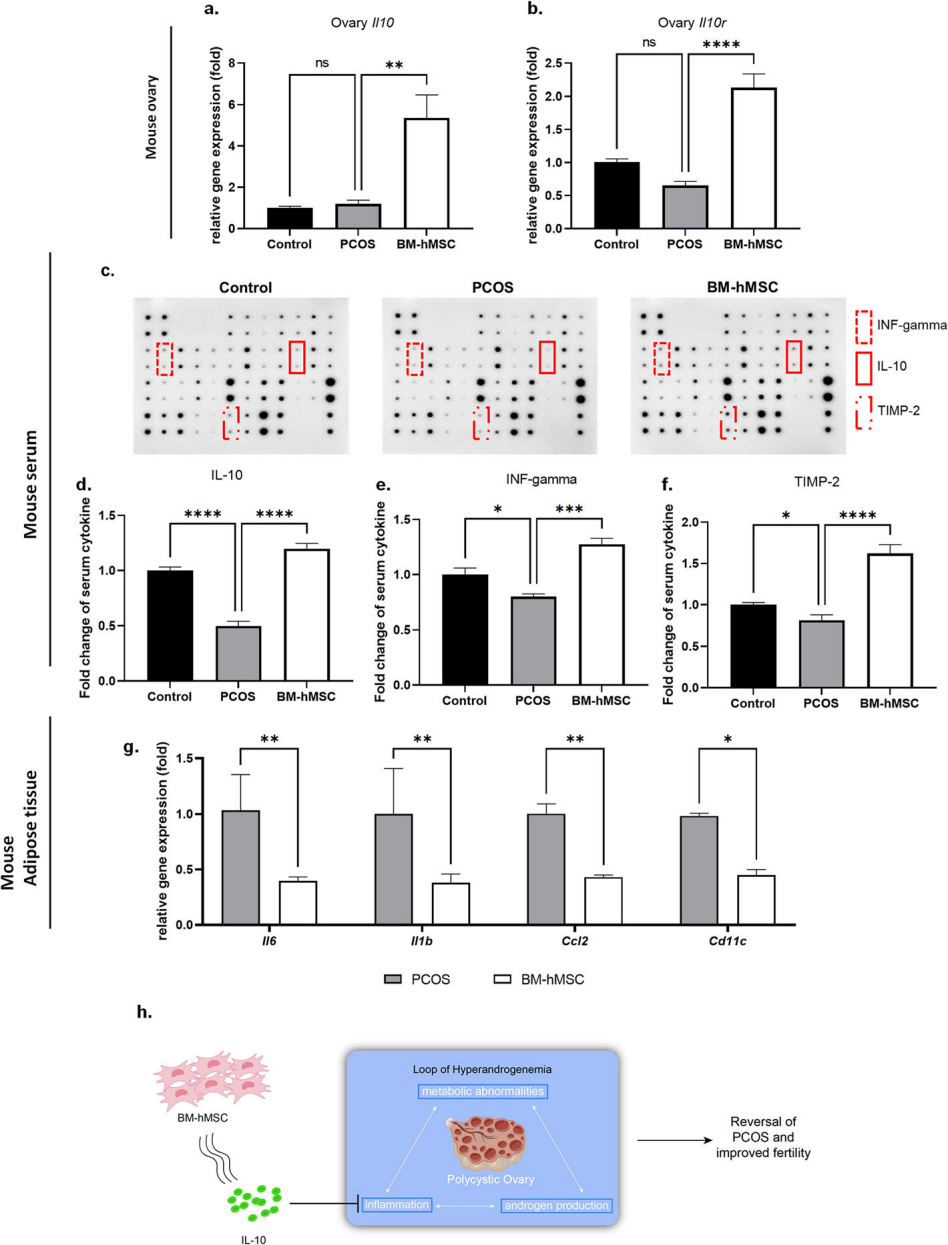
Effect of BM-hMSC on cytokines in the LTZ-induced PCOS mouse model. Relative gene expression of **a** IL-10 (*Il10*) and **b** IL-10R (*Il10r*) in the ovary from control, untreated PCOS, and BM-hMSC-treated PCOS mice. **c** Serum levels of inflammatory regulatory markers by antibody-based membrane assay. Fold change of serum **d** IL-10, **e** INF-γ, and **f** TIMP-2 in control, PCOS, and BM-hMSC-treated PCOS mice. **g** Pro-inflammation marker mRNA expression of IL-6 (*Il6*), IL-1β (*Il1b*), CCL2 (*Ccl2*), and CD11c (*Cd11c*) in untreated and BM-hMSC-treated PCOS mice adipose tissue. **h** Schematic of the proposed model for BM-hMSC therapeutic effect in PCOS. A positive stimulation loop between inflammation, androgen production, and metabolic abnormalities could lead to PCOS. BM-hMSC alleviate the inflammation via secretion of anti-inflammatory factor IL-10, which suppresses androgen secretion by ovarian theca cells. Those effects in turn can improve the metabolic abnormalities. Regulation of inflammation through IL-10 leads to improved fertility in PCOS. *p<0.05, **p<0.005; NS, not significant. All graphs are presented as the mean ± SD (n≥3). Statistical significances were determined by 2-way ANOVA or nonparametric T-test (Mann-Whitney test) using GraphPad Prism 9

Our data showed that BM-hMSC engraftment reverses several key PCOS-related features such as insulin resistance, increased expression of androgen synthesis genes, a pro-inflammatory milieu, and abrogated fat metabolism. Notably, insulin resistance, androgen synthesis, and fat metabolism are all correlated with inflammation [57]. We explored whether the effects of BM-hMSC treatment are mediated by anti-inflammatory factors within its secretome, such as IL-10. We first analyzed ovarian *Il10* gene expression in all experimental groups. Interestingly, *Il10* gene expression in ovary tissue was significantly higher in BM-hMSC-treated PCOS ovaries (5.37 ± 2.72 fold) compared with untreated PCOS ovaries (1.19 ± 0.46 fold; Fig. 6a). Moreover, IL-10 receptor gene (*Il10r*) expression in ovary tissue was also significantly higher in the BM-hMSC-treated PCOS group (2.13 ± 0.57 fold) compared with the untreated PCOS group (0.65 ± 0.17 fold; Fig. 6b). Several reports have demonstrated an increased pro-inflammatory milieu in fat tissues of PCOS women [58] and animal models [59]. We assessed the impact of intra-ovarian delivery of BM-hMSC on white gonadal fat tissue inflammatory markers using qPCR. BM-hMSC treatment significantly downregulated *Il6* (0.39 ± 0.04 fold), *Il1b* (1.0 ± 0.41 fold), *Ccl2* (0.43 ± 0.02 fold), and *Cd11c* (0.45 ± 0.05 fold) expression in the fat tissue of BM-hMSC-treated PCOS mice versus that of untreated PCOS mice (Fig. 6g). Several inflammatory regulators, such as IL-10, IFN-γ, and TIMP-2, have been found to be lower in PCOS patients compared with healthy women [42, 60–62]. We tested mouse serum using an antibody-based membrane assay and found that these cytokines were significantly lower in the untreated PCOS group (IL-10: 0.50 ± 0.08 fold, INF-γ: 0.79 ± 0.05 fold, TIMP-2: 0.82 ± 0.13 fold) compared with control mice (Fig. 6c). Importantly, these cytokines were significantly increased in PCOS mice after BM-hMSC treatment (IL-10: 1.20 ± 0.09 fold, INF-γ: 1.27 ± 0.11 fold, TIMP-2: 1.62 ± 0.22 fold; Fig. 6d–f). These results suggest that intra-ovarian injection of BM-hMSC has a systemic anti-inflammatory effect in the PCOS mouse model, likely mediated by IL-10 secretion from these cells.

Taken together, our data suggests that intra-ovarian injection of BM-hMSC reduces inflammation by increasing the expression of anti-inflammatory mediators such as IL-10 and its receptor in the ovary, and circulating IL-10, IFN-γ, and TIMP-2 in serum, while decreasing pro-inflammatory mediators such as IL-6, IL-1β, CCL2, and CD11c gene expression in periovarian adipose tissue.

## Discussion

In this study, we report a significant inhibitory effect of the BM-hMSC secretome on steroidogenesis gene expression, inflammation, and androgen production in H295R cells, as well as in primary cultures of theca cells from women with PCOS. Additionally, our in vivo experimental data showed that intra-ovarian engraftment of BM-hMSC is capable of correcting several PCOS-related metabolic abnormalities in a mouse model of PCOS. While this LTZ-induced PCOS mouse model is infertile [19], we demonstrated that BM-hMSC treatment was able to restore fertility and treated mice delivered healthy pups. Interestingly, similar improvements in metabolic and reproductive endpoints were achieved with injection of BM-hMSC secretome, suggesting that most, if not all, of BM-hMSC effects in this model are paracrine in nature.

Due to the limited availability of human PCOS theca cells, in this study, we used only two human PCOS theca cells samples. Unfortunately, this small sample size may not be enough to represent every PCOS patients with various phenotypes. It would be very interesting if we analyze more human theca cells from various subtypes of PCOS patients.

Chronic inflammation plays an important role in PCOS pathogenesis [32]. BM-hMSC engraftment significantly reduced several inflammatory markers in PCOS mouse ovaries. Reports have demonstrated a positive feedback loop between inflammation and androgen production [11, 12, 32], suggesting that androgen synthesis and inflammation could be reciprocally self-propagated. Upregulation of CYP17A1 gene expression through oxidative stress, which is a known stimulator of inflammation [63, 64], also demonstrates a positive feedback loop in PCOS. Surprisingly, while there was significant suppression of androgen production in vitro after BM-hMSC secretome treatment, we did not see a difference in serum testosterone levels between the untreated PCOS and BM-hMSC-treated PCOS groups. This could be attributed to the episodic nature of steroid hormone secretion. Furthermore, our findings may highlight a limitation of the chemically (LTZ)-induced PCOS model, which primarily relies on the induction of higher testosterone accumulation via marked supraphysiological inhibition of its aromatization [19]. Key ovarian steroidogenic genes as *Cyp17a1* were upregulated in the PCOS group and significantly suppressed by BM-hMSC treatment. The effect of engrafted BM-hMSC on ovarian cells could occur via cell-to-cell contact or paracrine effects through secreted humoral factors.

IL-10 is an important immune-suppressive and anti-inflammatory cytokine that is key to several human disorders, including PCOS. Recent reports showed significantly lower serum levels of IL-10 in PCOS women compared with age- and BMI-matched healthy controls [42]. BM-hMSC secrete physiologically relevant quantities of IL-10 [31, 39, 40], which we confirmed in the BM-hMSC used in this work (Fig. 2a). It is well documented that PCOS patients exhibit significantly lower serum levels of IL-10 compared to healthy counterparts [42, 65, 66]. This triggered us to focus on this important immune-suppressive cytokine. Our working hypothesis then was that the low level of IL-10 in PCOS patients can be corrected by IL-10 secreted by transplanted MSCs via paracrine fashion. We showed that IL-10 treatment significantly downregulates steroidogenesis and inflammatory gene expression as well as suppresses androgen production by H295R cells (Fig. 2f). In vivo, BM-hMSC treatment significantly increased IL-10 expression in ovarian tissue and its serum concentration in PCOS mice. These results suggest that BM-hMSC can ameliorate PCOS-induced inflammation through IL-10 secretion, and IL-10-overexpressing BM-hMSC might be a novel and robust therapeutic approach for PCOS treatment (Fig. 6h).

The published studies reported that both TIMP-2 and IFN-gamma are decreased in PCOS patients [60, 61]. Our serum analysis in our mouse model also confirmed that these two immunomodulatory factors were decreased in PCOS mice and successfully reversed after MSC treatment. Detailed study of other secreted factors by MSC in PCOS condition will be addressed in future investigation.

The method of successful intra-ovarian injection of MSCs was previously established in our lab. In our previous study, we showed the presence of injected human MSC cells in to the ovarian tissue by immune fluorescence assay [67]. Our recent study also suggested that engrafted MSC by intra-ovarian injection stayed in to the ovary for more than 2 weeks [68]. Our PCR-based quantification analysis for engrafted human MSC shows that approximately 30% of cells are still present in the ovary for 2 weeks after injection and later gradually degraded [68]. Due to limited lifespan of the engrafted MSC, it is not possible to expect a long-term treatment by only single injection of MSC. However, the short lifespan and gradually degradation of engrafted cells might be beneficial for the safety point of view using allogenic stem cell-based therapy. Further study needs to enhance the therapeutic effect of MSC by isolating the therapeutic factor to modify the cells for the use of long-term treatment including patient derived clinical trials.

Recently, Mehri Azadbakht group and Lamei Cheng group published papers that MSC treatment in PCOS mouse models was able to reduce immune response [69, 70]. Both papers reported that altered pro-inflammatory cytokines TNF-α in PCOS mice was significantly decreased after MSC treatment in the serum level and ovary gene expression. In addition, Lamei Cheng’s paper [70] shows high percentages of the peripheral and splenic neutrophils (Ly6G+CD11b+) and macrophages (F4/80+) in PCOS mouse model were decreased as normal level after MSC treatment. Moreover, in their peripheral blood flow cytometry data, they also revealed that the administration of MSC significantly reduced the percentage of pro-inflammatory M1 macrophages (CD11c+) and increased the percentage of anti-inflammatory M2 macrophages (CD206+) compared with PCOS mouse model. These results are consistent with an immune modulatory effect of MSC therapy. Detailed immuno-histochemistry and infiltration of various immune cells in the ovaries will be performed in future studies.

Although we demonstrate the various therapeutic effect of hMSC in PCOS, our study still has some limitations. First is small sample size for human PCOS patient samples. Unfortunately, it is very hard to get a human PCOS patient theca cell. Although we tested with androgen-producing H295R cells and PCOS mouse model, the effect of MSC treatment on actual PCOS patient could be differ compared to our data. Second is lack of direct evidence of MSC engraftment and inflammatory infiltration in the ovary tissue. In our previous study, we already reported that engrafted hMSCs are found in the ovary [67] and stayed in ovary more than 2 weeks [68]. Other previous studies also reported that decreased population of immune cells [70]. Although descriptions of these published studies, we cannot rely to a high extent on previous data to explain our data. Due to this limitation, our data suggesting a possible regulatory pathway through some markers but still not clear the effect on the outcome of inflammation through immune cells itself. In our further study, we need to analyze the effect on immune cells infiltration and distribution of injected factors.

Recently, two reports have described the utility of tail-vein injected MSCs to reverse some PCOS immune-phenotypes [69, 70]. In these studies, MSCs inhibited T cell proliferation, decreased inflammation in vitro and in vivo, and enhanced ovarian function in a PCOS animal model. However, these studies lacked translational fertility, reproductive, and metabolic outcomes data. Moreover, many reports have described that, after systemic infusion, stem cells are trapped in the lungs with limited in vivo persistence [71–73]. In contrast, local engraftment of hMSCs directly into the target organ, as we describe here, can initiate the reparative process in a more robust manner for cell homing and effective tissue repair [74]. Additional research is needed to evaluate the utility of intra-ovarian engraftment of BM-hMSC as a potentially promising approach for the treatment of PCOS-associated infertility in women.

## Conclusions

Polycystic ovary syndrome (PCOS) is the most common disease in women. Many PCOS patient typically shows excessive inflammation and infertility. In this study, we present a stem cell-based therapy to restore fertility in PCOS condition. We report that BM-hMSC secretome inhibit steroidogenesis, inflammation, and androgen production in both H295R cells and primary cultured theca cell. We also report the therapeutic efficacy of mesenchymal stem cells (BM-hMSC) in PCOS mouse models. Our study shows that BM-hMSC can treat PCOS-related characteristics, including infertility. This approach may represent a novel therapeutic option for women with PCOS.

## Supplementary Information

**Additional file 1: Figure S1.** Human bone marrow mesenchymal stem cells (BM-hMSC) characterization. **Figure S2.** BM-hMSC decrease expression of steroidogenesis genes in Forskolin-treated human PCOS theca cells. **Figure S3.** Timeline for in vivo experimental design. **Figure S4.** BM-hMSC reverse brown fat tissue phenotype in the LTZ-induced PCOS mouse. **Figure S5.** BM-hMSC reverse adipose tissue adipokines in LTZ-induced PCOS mouse model. **Figure S6.** Hormonal analysis in the LTZ-induced PCOS mouse model. **Figure S7.** The effect of BM-hMSC on the endometrium in the LTZ-induced PCOS mouse model. **Figure S8.** Effect of BM-hMSC secretome injection on the ovary, white fat, brown fat, and fertility in the LTZ-induced PCOS mouse model. **Figure S9.** Raw data image of Western blot membranes. **Table S1.** List of Primers.

## Data Availability

Not applicable

## Abbreviations

BM-hMSC: Bone marrow derived human mesenchymal stem cell
PCOS: Polycystic ovary syndrome
LTZ: Letrozole
IHC: Immunohistochemistry
qRT-PCR: Quantitative real-time polymerase chain reaction
IL-1β: Interleukin-1 beta
IL-10: Interleukin-10
TNF-α: Tumor necrosis factor
MSCGM: Mesenchymal stem cell growth medium
FBS: Fetal bovine serum
PBS: Phosphate-buffered saline
UIC ACC: University of Illinois at Chicago Animal Care Committee
CLAMS: Comprehensive Lab Animal Monitoring System
VO_2_: Oxygen consumption rate
VCO_2_: Carbon dioxide release
RER: Respiratory exchange ratio
T: Testosterone
E2: Estradiol
LH: Luteinizing hormone
FSH: Follicle-stimulating hormone
H&E: Hematoxylin-eosin
