# Supplementary material for: Mesenchymal stem cell therapy ameliorates metabolic dysfunction and restores fertility in a PCOS mouse model through interleukin-10
Source: Stem Cell Res Ther. 2021 Jul 7;12:388. doi: 10.1186/s13287-021-02472-w (PMC8261924; doi:10.1186/s13287-021-02472-w)
Supplement: Supplementary file 1 — Additional file 1: Figure S1. Human bone marrow mesenchymal stem cells (BM-hMSC) characterization. Figure S2. BM-hMSC decrease expression of steroidogenesis genes in Forskolin-treated human PCOS theca cells. Figure S3. Timeline for in vivo experimental design. Figure S4. BM-hMSC reverse brown fat tissue phenotype in the LTZ-induced PCOS mouse. Figure S5. BM-hMSC reverse adipose tissue adipokines in LTZ-induced PCOS mouse model. Figure S6. Hormonal analysis in the LTZ-induced PCOS mouse model. Figure S7. The effect of BM-hMSC on the endometrium in the LTZ-induced PCOS mouse model. Figure S8. Effect of BM-hMSC secretome injection on the ovary, white fat, brown fat, and fertility in the LTZ-induced PCOS mouse model. Figure S9. Raw data image of Western blot membranes. Table S1. List of Primers. [file 13287_2021_2472_MOESM1_ESM.docx]

**Supplementary Materials**

Fig S1: Human bone marrow mesenchymal stem cells (BM-hMSC) characterization.

Fig S2: BM-hMSC decrease expression of steroidogenesis genes in Forskolin-treated human PCOS theca cells.

Fig S3: Timeline for in vivo experimental design.

Fig S4: BM-hMSC reverse brown fat tissue phenotype in the LTZ-induced PCOS mouse.

Fig S5: BM-hMSC reverse adipose tissue adipokines in LTZ-induced PCOS mouse model.

Fig S6: Hormonal analysis in the LTZ-induced PCOS mouse model.

Fig S7: The effect of BM-hMSC on the endometrium in the LTZ-induced PCOS mouse model.

Fig S8: Effect of BM-hMSC secretome injection on the ovary, white fat, brown fat, and fertility in the LTZ-induced PCOS mouse model.

Fig S9: Raw data image of Western blot membranes

Table S1. List of Primers

**Supplementary Materials**

**
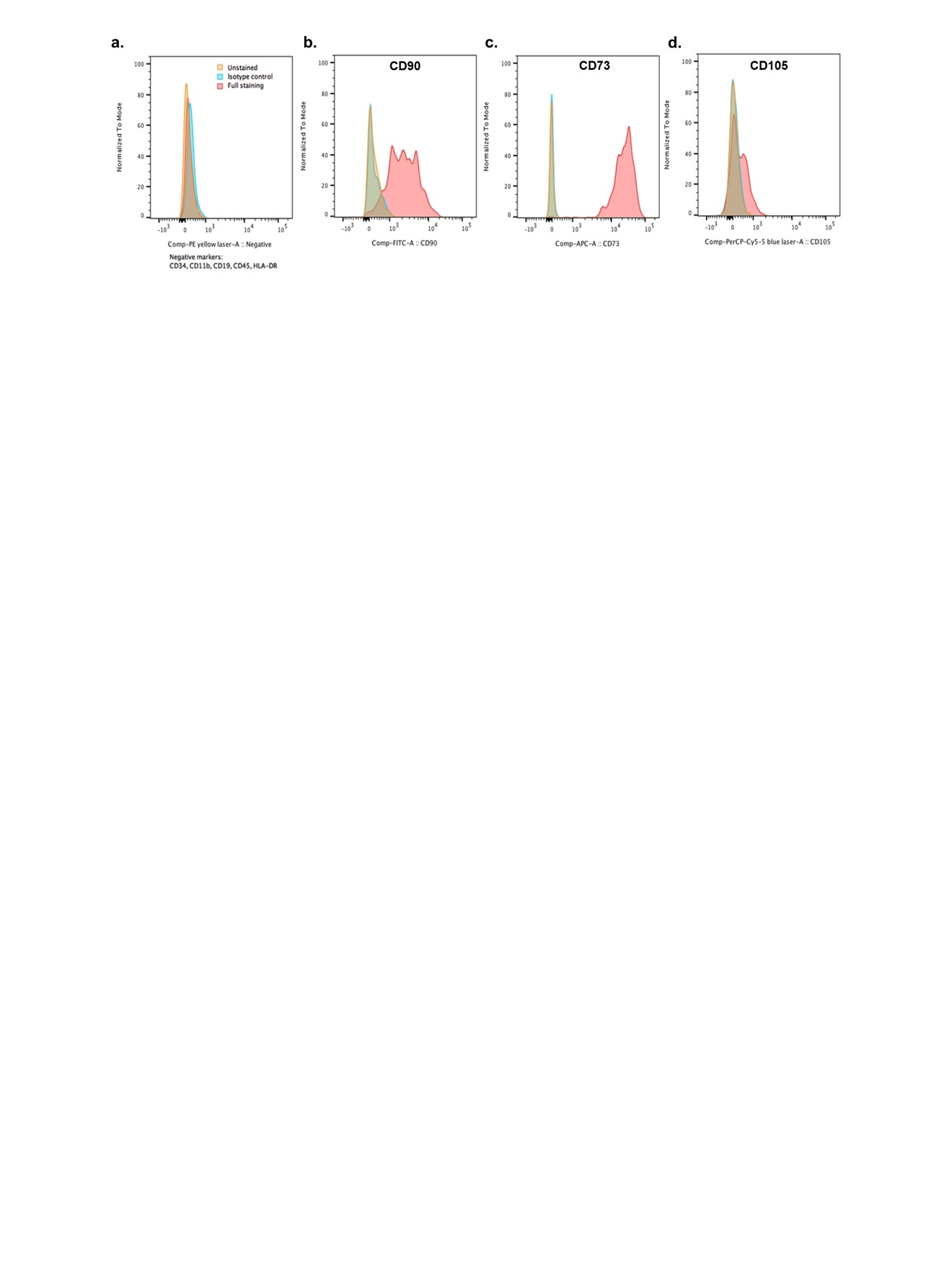
**

***Fig S1: Human bone marrow mesenchymal stem cells (BM-hMSC) characterization.*** *BM-hMSC were isolated from a 32-year-old healthy adult female bone marrow (non-diabetic). Cells were characterized by* ***(a)*** *lineage negative for CD11b, CD34, CD19, CD45, and HLA-DR, and lineage positive for* ***(b)*** *CD90,* ***(c)*** *CD73, and* ***(d)*** *CD105.*

*
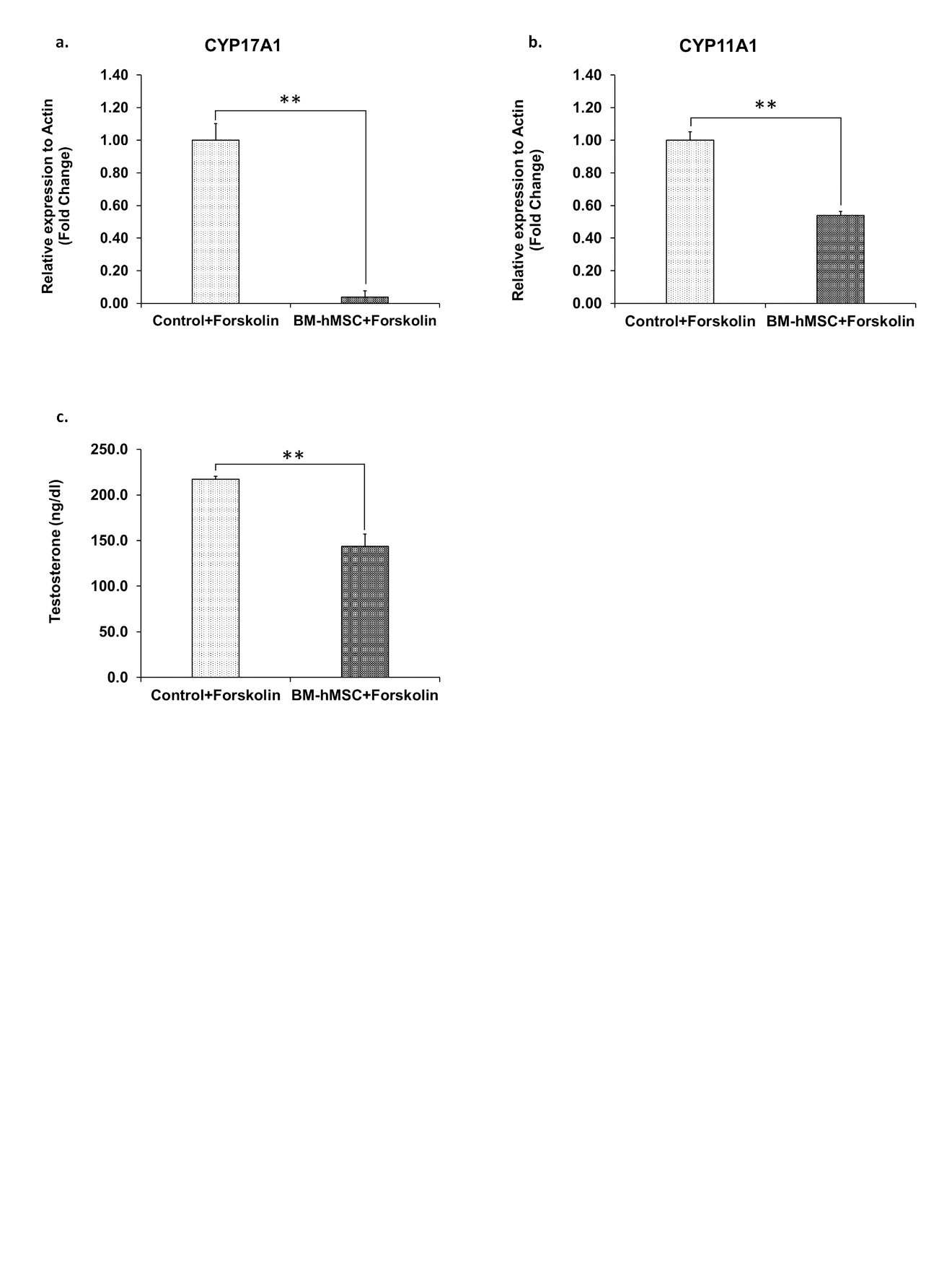
*

***Fig S2:*** ***BM-hMSC decrease expression of steroidogenesis genes in Forskolin-treated human PCOS theca cells. (a)*** *Relative mRNA expression of CYP17A1 and* ***(b)*** *CYP11A1 in Forskolin-treated human PCOS theca cells (n=3) after BM-hMSC secretome treatment.* ***(c)*** *Testosterone secretion by Forskolin-treated human PCOS theca cells; BM-hMSC secretome vs. control group. *: p<0.05, **: p<0.005*


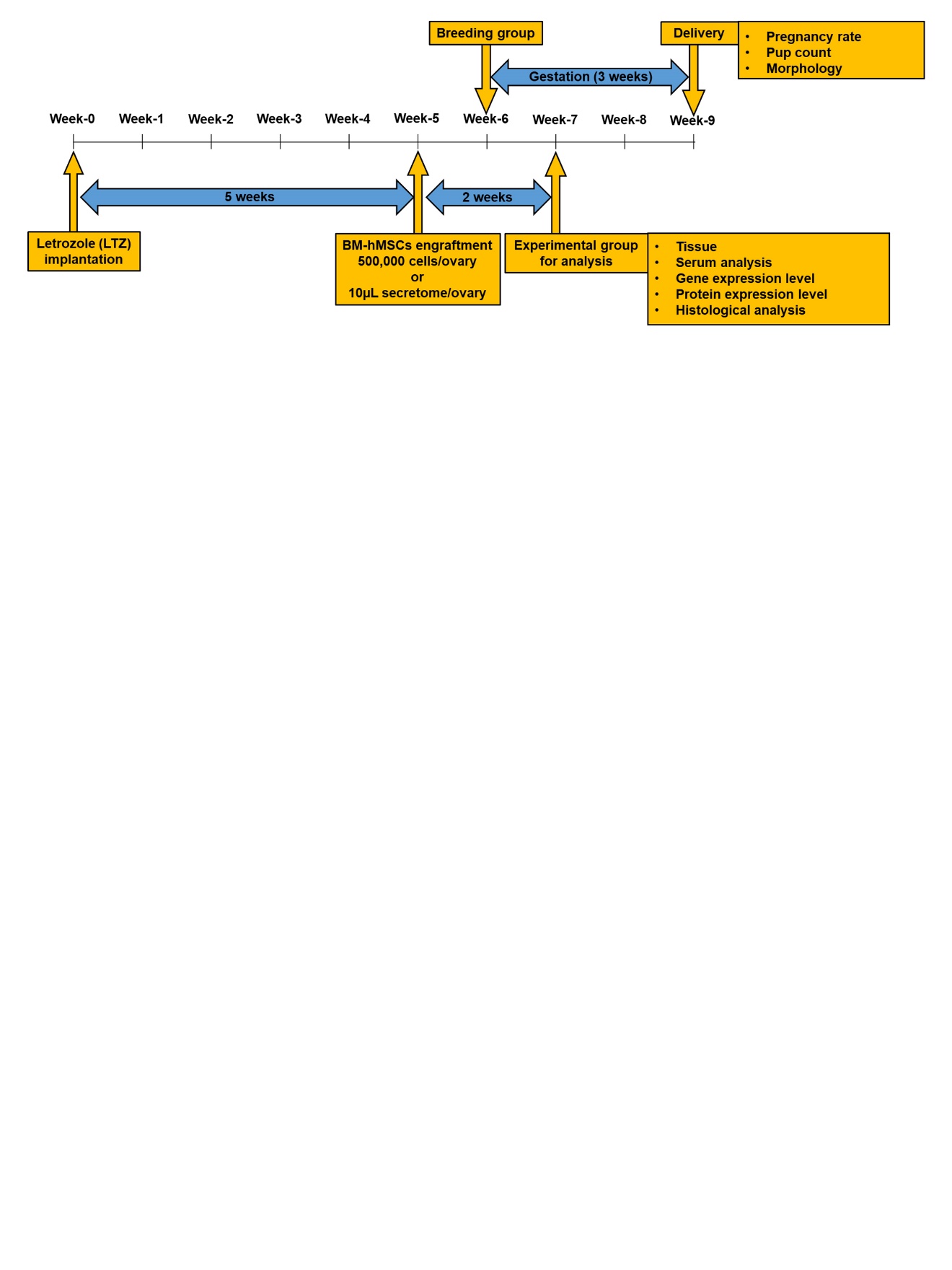


***Fig S3: Timeline for in vivo experimental design.***


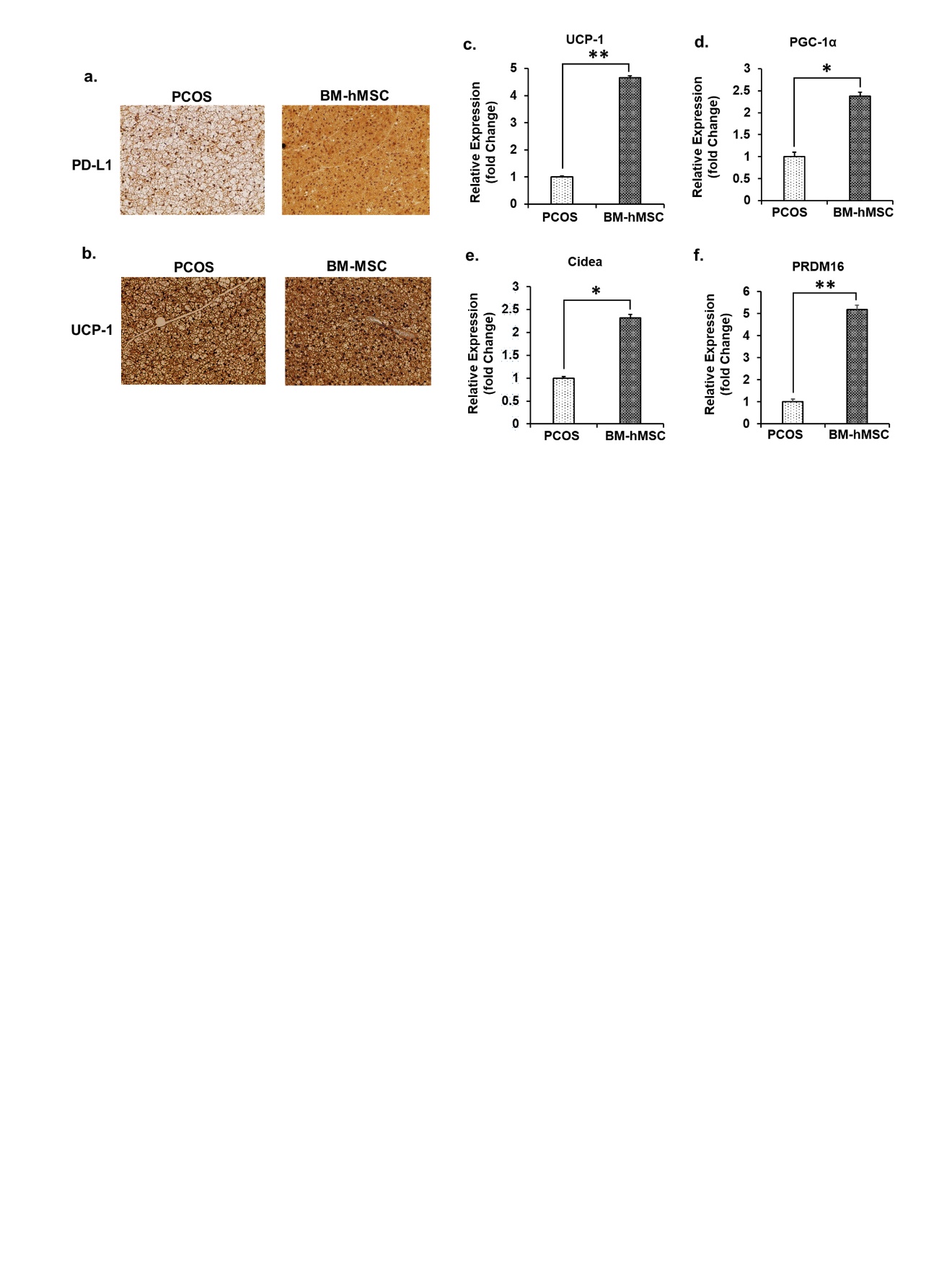


***Fig S4:*** ***BM-hMSC reverse brown fat tissue phenotype in the LTZ-induced PCOS mouse.*** *Immunohistochemistry of brown fat tissue by* ***(a)*** *PD-L1 and* ***(b)*** *UCP-1.* ***(c)*** *Relative gene expression of UCP-1 in brown fat of PCOS mice (1.0 ± 0.03 fold) and BM-hMSC-treated PCOS mice (4.66 ± 0.07 fold).* ***(d)*** *Relative gene expression of PGC-1α in brown fat of PCOS mice (1.0 ± 0.10 fold) and BM-hMSC-treated PCOS mice (2.38 ± 0.09 fold).* ***(e)*** *Relative gene expression of Cidea in brown fat of PCOS mice (1.0 ± 0.04 fold) and BM-hMSC-treated PCOS mice (2.31 ± 0.08 fold).* ***(f)*** *Relative gene expression of PRDM16 in the brown fat of PCOS mice (1.0 ± 0.12 fold) and BM-hMSC-treated PCOS mice (5.19 ± 0.19 fold). Data presented as the mean ± SD. *: p<0.05, **: p<0.005.*

**
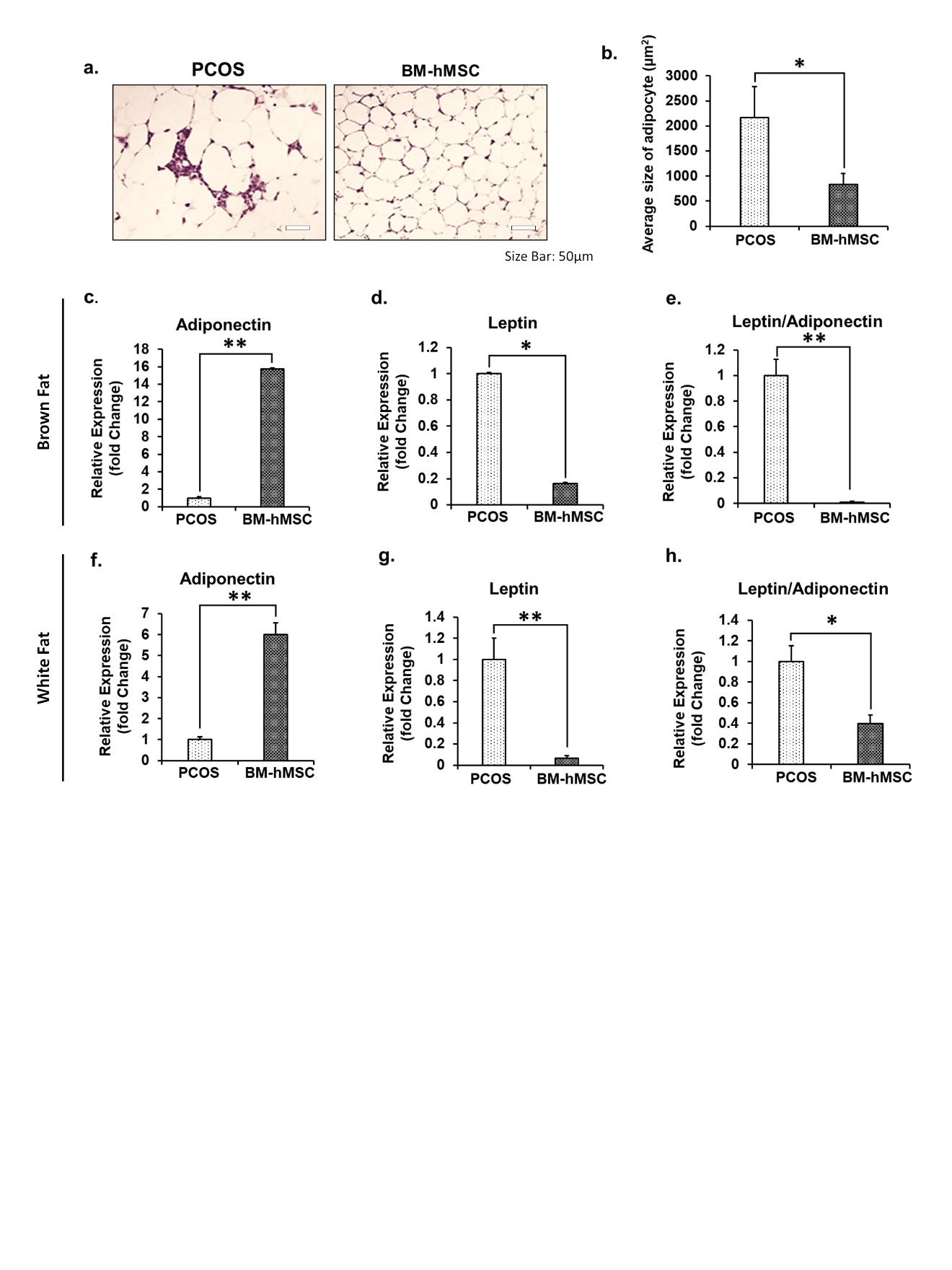
**

***Fig S5:*** ***BM-hMSC reverse adipose tissue adipokines in LTZ-induced PCOS mouse model.*** ***(a)*** *H&E staining of white gonadal fat, arrows indicate immune cells infiltrating adipocyte interstitial space in PCOS mice treated with BM-hMSC and untreated PCOS mice.* ***(b)*** *The average size of adipocytes in PCOS group (2172.7 ± 610.5 µm^2^) and BM-hMSC-treated PCOS group (828.7 ± 223.1 µm^2^).* ***(c-h)*** *Real-time PCR of brown and white gonadal fat comparing untreated PCOS group and BM-hMSC-treated PCOS group.* ***(c)*** *Relative gene expression of adiponectin in brown fat of PCOS mice (1.0 ± 0.13 fold) and BM-hMSC-treated PCOS mice (15.75 ± 0.12 fold).* ***(d)*** *Relative gene expression of leptin in brown fat of PCOS mice (1.0 ± 0.01 fold) and BM-hMSC-treated PCOS mice (0.16 ± 0.01 fold).* ***(e)*** *Ratio of leptin to adiponectin in the brown fat of PCOS mice (1.0 ± 0.13 fold) and BM-hMSC-treated mice (0.01 ± 0.01 fold).* ***(f)*** *Relative gene expression of adiponectin in white fat of PCOS mice (1.0 ± 0.14 fold) and BM-hMSC-treated mice (6.00 ± 0.57 fold).* ***(g)*** *Relative gene expression of adiponectin in white fat of PCOS mice (1.0 ± 0.20 fold) and BM-hMSC-treated mice (0.07 ± 0.02 fold).* ***(h)*** *Ratio of leptin to adiponectin in white fat of PCOS mice (1.0 ± 0.15 fold) and BM-hMSC-treated PCOS mice (0.4 ± 0.08 fold). Data presented as the mean ± SD. *: p<0.05, **: p<0.005.*

***
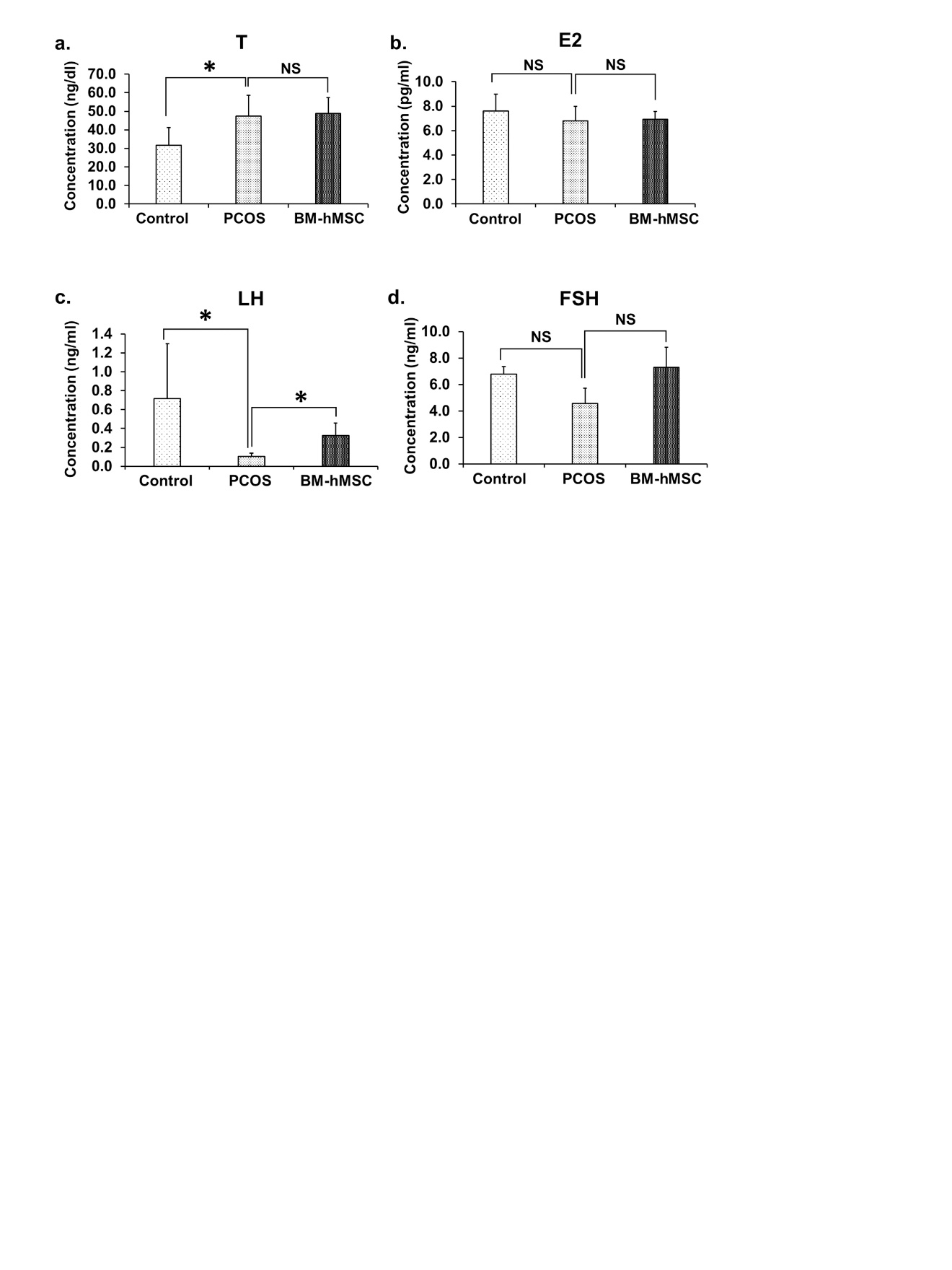
***

***Fig S6: Hormonal analysis in the LTZ-induced PCOS mouse model.*** *(a-d) Average serum hormone levels (n=8/group).* ***(a)*** *Level of serum testosterone (T) in control (31.8 ± 9.5 ng/dL), PCOS (47.3 ± 11.4 ng/dL), and BM-hMSC-treated PCOS groups (48.8 ± 8.4 ng/dL).* ***(b)*** *Level of serum estradiol (E2) in control (7.6 ± 1.4 pg/dL), PCOS (6.8 ± 1.2 pg/dL), and BM-hMSC-treated PCOS groups (6.9 ± 0.63 pg/dL).* ***(c)*** *Level of serum luteinizing hormone (LH) in control (0.7 ± 0.58 ng/dL), PCOS (0.1 ± 0.03 ng/dL), and BM-hMSC-treated PCOS groups (0.3 ± 0.13 ng/dL).* ***(d)*** *Level of serum follicle-stimulating hormone (FSH) in control (6.8 ± 0.6 ng/dL), PCOS (4.6 ± 1.1 ng/dL), and BM-hMSC-treated PCOS groups (7.3 ± 1.5 ng/dL). Data presented as the mean ± SD. *: p<0.05; NS: Not significant.*


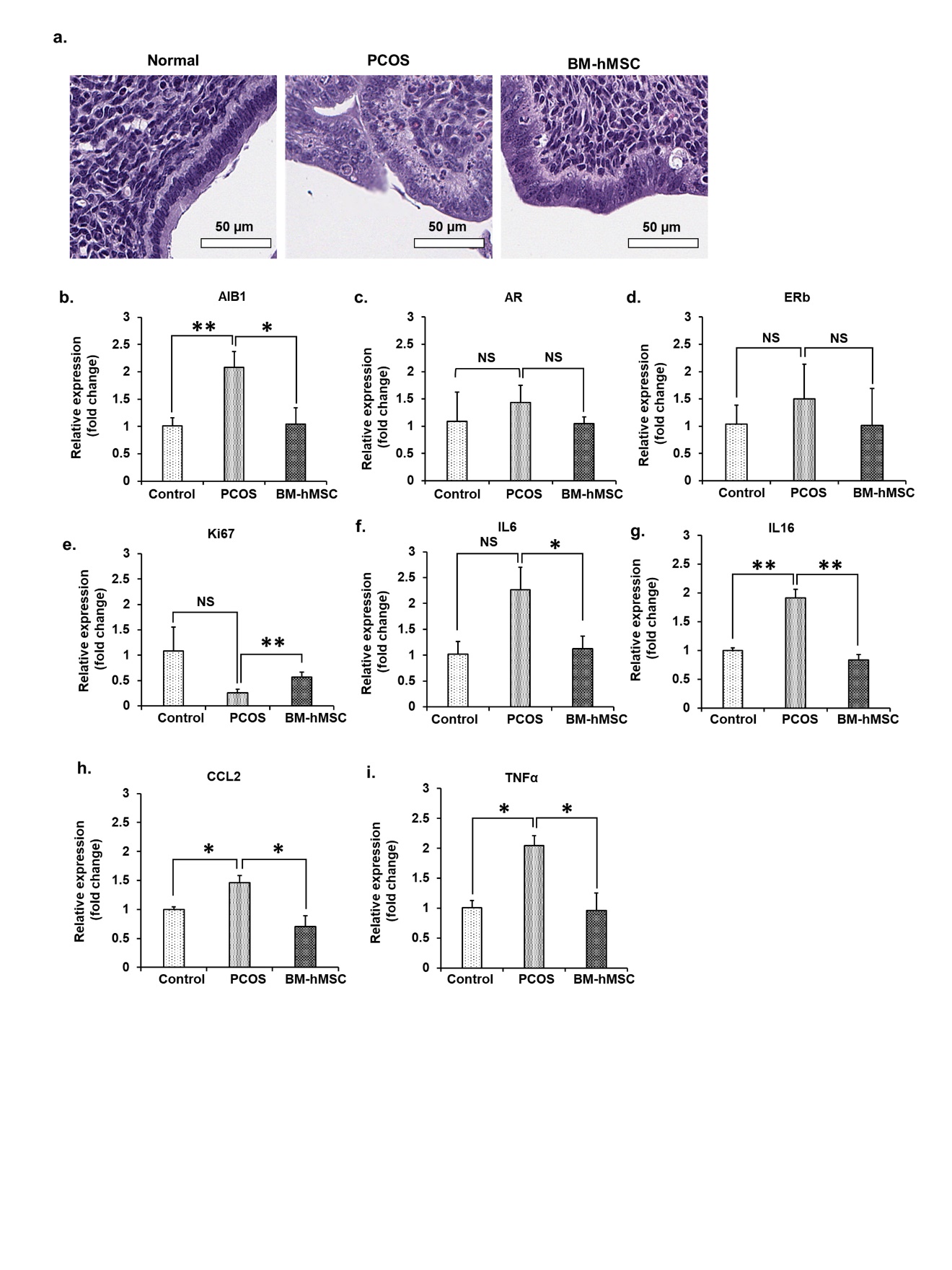


***Fig S7: The effect of BM-hMSC on the endometrium in the LTZ-induced PCOS mouse model.*** ***(a)*** *Morphology of mouse endometrium tissue in control, PCOS, and BM-hMSC-treated PCOS mice (H&E staining).* ***(b)*** *Comparison of relative AIB1 gene expression level in control (1.00 ± 0.15 fold), PCOS (2.08 ± 0.29 fold), BM-hMSC-treated PCOS (1.04 ± 0.30 fold) mouse endometrium.* ***(c)*** *Comparison of relative AR gene expression level in control (1.08 ± 0.54 fold), PCOS (1.44 ± 0.31 fold), and BM-hMSC-treated PCOS (1.05 ± 0.12 fold) mouse endometrium.* ***(d)*** *Comparison of relative ER*β *gene expression level in control (1.03 ± 0.35 fold), PCOS (1.50 ± 0.63 fold), and BM-hMSC-treated PCOS (1.01 ± 0.68 fold) mouse endometrium.* ***(e)*** *Comparison of relative Ki67 gene expression level in control (1.08 ± 0.47 fold), PCOS (0.26 ± 0.07 fold), and BM-hMSC-treated PCOS (0.57 ± 0.09 fold) mouse endometrium.* ***(f)*** *Comparison of relative IL6 gene expression level in control (1.02 ± 0.24 fold), PCOS (2.26 ± 0.44 fold), and BM-hMSC-treated PCOS (1.13 ± 0.24 fold) mice endometrium.* ***(g)*** *Comparison of relative IL16 gene expression level in control (1.00 ± 0.04 fold), PCOS (1.91 ± 0.14 fold), and BM-hMSC-treated PCOS (0.83 ± 0.09 fold) mouse endometrium.* ***(h)*** *Comparison of relative CCL2 gene expression level in control (1.00 ± 0.04 fold), PCOS (1.46 ± 0.12 fold), and BM-hMSC-treated PCOS (0.70 ± 0.19 fold) mouse endometrium.* ***(i)*** *Comparison of relative TNFα gene expression level in control (1.00 ± 0.12 fold), PCOS (2.05 ± 0.16 fold), and BM-hMSC-treated PCOS (0.97 ± 0.29 fold) mouse endometrium. Data presented as the mean ± SD. *: p<0.05, **: p<0.005, ***: p<0.0005; NS: Not significant.*

**
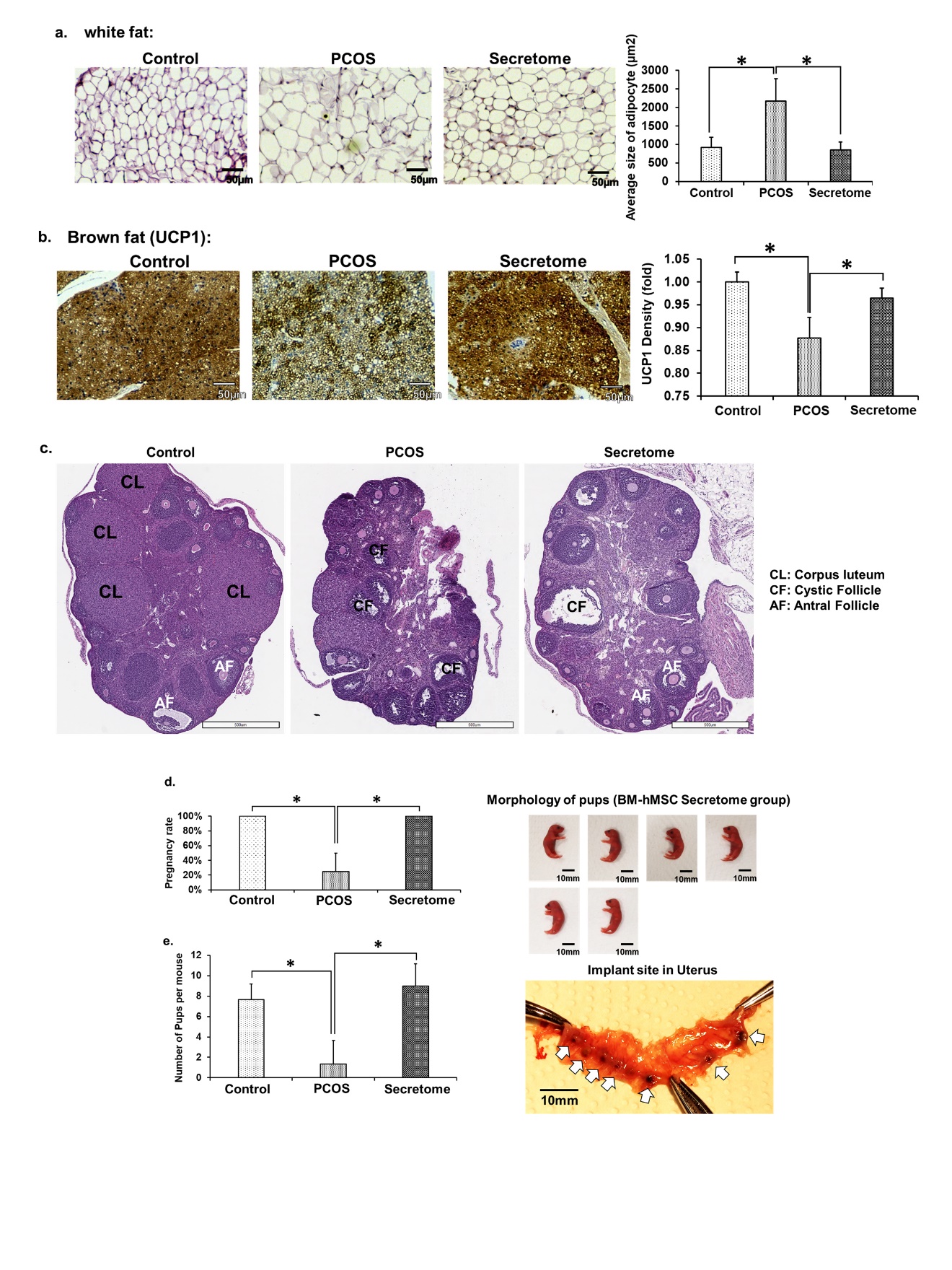
**

***Fig S8: Effect of BM-hMSC secretome injection on the ovary, white fat, brown fat, and fertility in the LTZ-induced PCOS mouse model****.* ***(a)*** *Morphology of ovary with H&E staining in control mice (Control), PCOS mice (PCOS), and BM-hMSC secretome-treated PCOS mice (Secretome).* ***(b)*** *Size of adipocyte in white fat tissue from the control group (926 ± 266 µm2), PCOS group (2173 ± 610 µm2), and Secretome group (829 ± 223 µm2).* ***(c)*** *Density of brown fat analyzed with UCP1 by immunohistochemistry.* ***(d)*** *Pregnancy rate of the control group (4 out of 4), the PCOS group (1 out of 4), and the Secretome group (4 out of 4).* ***(e)*** *Average number of pups from the control group (7.6 ± 1.5), PCOS group (1.3 ± 2.3), and the Secretome group (9.0 ± 2.2). Data presented as the mean ± SD. *: p<0.05.*

**
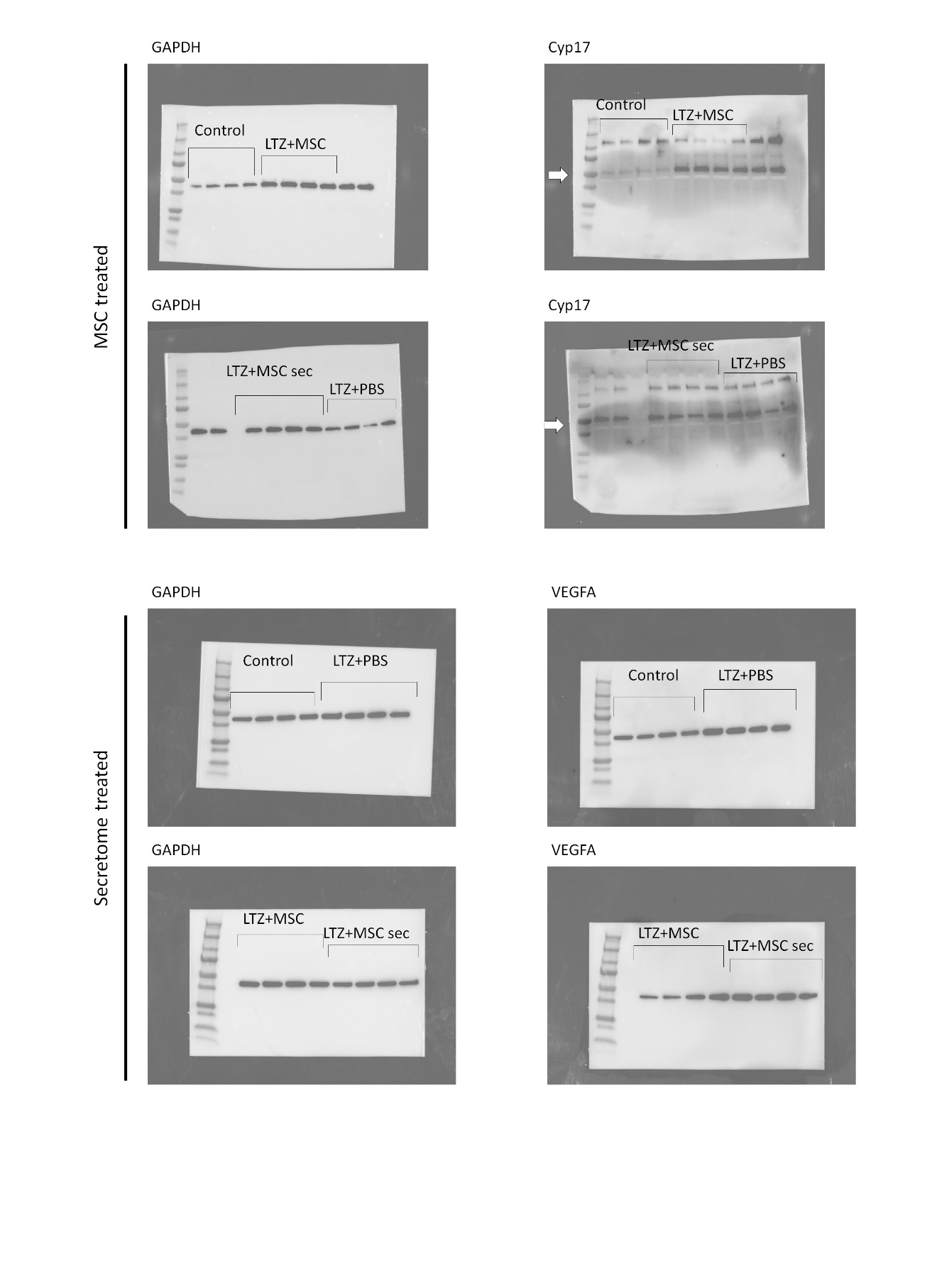
**

***Fig S9: Raw data image of Western blot membranes***

**Table S1. List of Primers**

| **Gene** | **Forward primer (5’-3’)** | **Reverse primer (5’-3’)** |
| --- | --- | --- |
| Human *ACTB* | TGGATCAGCAAGCAGGAGTATG | GCATTTGCGGTGGACGAT |
| Human *CYP17A1* | GGCCTCAAATGGCAACTCTAGA | CTTCTGATCGCCATCCTTGAA |
| Human *CYP11A1* | GAGGGAGACGGGCACACA | TGACATAAACCGACTCCACGTT |
| Human *DENND1A* | CAATTCCCGGAGGACTACAGT | AGCACGAATGTGAAGTTCTGG |
| Human *IL6* | TGCACTTTATGACGCACTCAC | TGTCCAAAAACACGAAATCATGC |
| Human *IL1B* | ATGATGGCTTATTACAGTGGCAA | GTCGGAGATTCGTAGCTGGA |
| Human *TNFA* | AAGCACACTGGTTTCCACACT | TGGGTCCCTGCATATCCGTT |
| Mouse *Gapdh* | CACATTGGGGGTAGGAACAC | AACTTTGGCATTGTGGAAGG |
| Mouse *Cyp17a1* | GAGTTTGCCATCCCGAAGGA | CCAGCTCCGAAGGGCAAATA |
| Mouse *Cyp19a1* | TTTCGCTGAGAGACGTGGAG | AGGATTGCTGCTTCGACCTC |
| Mouse *Fshr* | GTGCATTCAACGGAACCCAG | TCTAAGCCATGGTTGGGCAG |
| Mouse *Vegfa* | GTACCTCCACCATGCCCAGT | GCATTCACATCTGCTGTGCT |
| Mouse *Il1b* | TTCAGGCAGGCAGTATCACTC | GAAGGTCCACGGGAAAGACAC |
| Mouse *Il6* | ATCCAGTTGCCTTCTTGGGACTGA | TAAGCCTCCGACTTGTGAAGTGGT |
| Mouse *Il10* | CGGGAAGACAATAACTGCACCC | CGGTTAGCAGTATGTTGTCCAGC |
| Mouse *Ccl2* | AGATGCAGTTAACGCCCCAC | ACCCATTCCTTCTTGGGGTC |
| Mouse *Cd11c* | ACGTCAGTACAAGGAGATGTTGGA | ATCCTATTGCAGAATGCTTCTTTACC |
| Mouse *Tnfa* | TCCCAGGTTCTCTTCAAGGG | GGTGAGGAGCACGTAGTCGG |
| Mouse *Ki67* | CTGCCTGCGAAGAGAGCATC | AGCTCCACTTCGCCTTTTGG |
| Mouse *Erb* | TTCCCGGCAGCACCAGTAACC | TCCCTCTTTGCGTTTGGACTA |
| Mouse *Ar* | TTGCAAGAGAGCTGCATCAGTT | ACTGTGTGTGGAAATAGATGGGC |
| Mouse *Aib1* | GCCTGGCTTTGAAGACATAATCCG | TCTTGATAGTGACGCTTCTGGGAC |
| Mouse *Adiponectin* | GGCCCATCATGCTATGGAAC | GTGAGGGATCACTCGCCATC |
| Mouse *Leptin* | TTGCCATATTCCTCACCA | CCCACTCGATCAGATGTGCTC |
| Mouse *Ucp1* | CAAAAACAGAAGGATTGCCGAAA | TCTTGGACTGAGTCGTAGAGG |
| Mouse *Pgc1a* | TATGGAGTGACATAGAGTGTG | GTCGCTACACCACTTCAATCC |
| Mouse *Cidea* | TGACATTCATGGGATTGCAGAC | CATGGTTTGAAACTCGAAAAGGG |
| Mouse *Prdm16* | CCACCAGCGAGGACTTCAC | GGAGGACTCTCGTAGCTCGAA |
